# Antiviral and ROS scavenging potential of *Carica papaya Linn* and *Psidium guajava* leaves extract against HIV-1 infection

**DOI:** 10.1186/s12906-023-03916-x

**Published:** 2023-03-18

**Authors:** Pratiksha Jadaun, Prachibahen Shah, R. Harshithkumar, Madhukar S. Said, Shubhangi P. Bhoite, Sowmya Bokuri, Selvan Ravindran, Neetu Mishra, Anupam Mukherjee

**Affiliations:** 1 ICMR-National AIDS Research Institute, Pune, 411026 MH India; 2 Symbiosis School of Biological Sciences, Symbiosis International Deemed University, Pune, 412115 MH India; 3 CSIR-National Chemical Laboratory, Pune, 411008 MH India

**Keywords:** Carica papaya Linn, Psidium guajava, HR-ESI–MS, Reactive oxygen species, Cytotoxicity, Anti-HIV-1 activity, Anti-retroviral, Bioactive constituents

## Abstract

Antiretroviral therapy is the only treatment option for HIV-infected patients; however, it has certain drawbacks in terms of developing multiple toxic side effects. Thus, there is a continuous need to explore safe and efficacious anti-retroviral agents. *Carica papaya* Linn and *Psidium guajava* are known for their various biological activities. In this study, we characterized the bioactive fractions of methanolic leaves extract from both plants using the High-resolution electrospray ionization mass spectrometry (HR-ESI–MS) technique, followed by the investigation of their potential as anti-HIV-1 and antioxidant agents through in vitro mechanistic assays. The anti-HIV-1 activity was examined in TZM-bl cells through luciferase gene assay against two different clades of HIV-1 strains, whereas the intracellular ROS generation was analyzed by Fluorescence-Activated Cell Sorting. Additionally, the mechanisms of action of these phyto-extracts were determined through the Time-of-addition assay. The characterization of *Carica papaya* Linn and *Psidium guajava* leaves extract through HR-ESI–MS fragmentation showed high enrichment of various alkaloids, glycosides, lipids, phenolic compounds, terpenes, and fatty acids like bioactive constituents. Both the phyto-extracts were found to be less toxic and exhibited potent antiviral activity against HIV-1 strains. Furthermore, the phyto-extracts also showed a decreased intracellular ROS in HIV-1 infected cells due to their high antioxidant potential. Overall, our study suggests the anti-HIV-1 potential of *Carica papaya* Linn and *Psidium guajava* leaves extract due to the synergistic action of multiple bioactive constituents.

**Supplementary Information:**

The online version contains supplementary material available at 10.1186/s12906-023-03916-x.

## Background

Human immunodeficiency virus (HIV), a member of the *Retroviridae* family in the genus of Lentivirus, is the main causing agent for severe immune suppression [1, 2]. The disease severity is responsible for the development of a chronic, deadly condition, called acquired immunodeficiency syndrome or AIDS, which commits lifelong threats to the infected individuals [3].

The employed existing antiretroviral therapies are showing promising results, but not enough for the complete eradication of HIV/AIDS [4]. Although ARTs have reduced the viral load, transmission, and morbidity/mortality ratio to a great extent, their mechanism of action showed deleterious effects related to mtDNA, impaired glucose metabolism, hepatotoxicity, multidrug-resistant strains after prolonged periods of infection and upsurge of the reactive oxygen species (ROS) within the cell [5–10]. Testing ethnomedicines and phytoconstituents for the development of anti-HIV potency like pharmaceuticals to overcome multidrug resistance, while at the same time, lacking in antioxidants is the need of the hour to meet the necessity of generating novel alternative treatments.

Studies have shown that plants are rich in secondary metabolites and phytochemicals like flavonoids, alkaloids, polyphenolics, sulphated polysaccharides, triterpenes, phenolics and coumarins. Moreover, phytoconstituent-based alternative medicines are cheaper, easily accessible, fewer side effects with better tolerance [11, 12]. Natural bioactive compounds that could possess a distinct mode of action are not usually found in synthetic drugs [13]. Various investigation has revealed the medicinal properties of phytotherapeutic plant *Carica papaya* Linn. (*C. papaya*) and *Psidium guajava* (*P. guajava*) as anti-virals, immunomodulatory, anti-inflammatory, antimicrobial, and antioxidant activity [14, 15]. The polyphenol compound of these plants such as tocopherol, ascorbic acid, carotenoids, folic acid and flavonoids are the strong biochemical antioxidant components [16]. Toxicological studies on mice and other animal models also showed no significant adverse effects, mutagenic, or aberrant effects of *C. papaya* and *P. guajava* [17–20].

Notably, there is very limited research available on the distinctive constituents of *C. papaya* and *P. guajava* that have shown any antiviral effects. Therefore, the present study was designed to examine the chemical compositions, antiviral properties, and antioxidant activities of these two methanolic phyto-extracts. It is known that the interaction between ROS and HIV infection may represent a new approach to both prevention and treatment, however, the effect of the methanolic extract of *C. papaya* and *P. guajava* has not been investigated extensively in the anti-HIV-1 research. Therefore, the present study was undertaken to characterize the leaves extract of *C. papaya* and *P. guajava* to identify the presence of bioactive components and assess the effectiveness of these phyto-extracts against HIV-1 infection and their antioxidant properties at the cellular level. We conducted cellular assays to examine the effects of these two plants extracts upon the pathogenicity of HIV-1 strains, unveiled the underlying mechanisms, and determined its role as a free radical scavenger during HIV-1 infection. Hence, this study might divulge the role of leaves extract of *C. papaya* and *P. guajava* as dual antiviral and cytoprotective anti-oxidant agents that might be helpful in the development of an novel anti-HIV-1 therapy or in conjunction with the present ART regime.

## Materials and methods

### Plant collection for phyto-extract preparation

The collection of plant material was done after obtaining the necessary permission, purely for research purposes and it complies with international and institutional guidelines and legislation. Briefly, fresh and young leaves of *C. papaya* and *P. guajava* were collected from the local region of the city, near Sinhagad Road, Pune, India. The voucher specimens were formally identified and deposited at the Western Regional Centre, Botanical Survey of India (BSI), Pune, India (authentication no. DP01 for *C. papaya* and NMPG-1 for *P. guajava*). The leaves were primarily washed under tap water followed by distilled water to remove the dust particles. Further, the clean leaves were shaded, dried and pulverized followed by the methanol extract preparation as described earlier [21]. Methanol is one of the effective solvents resulting in the highest extraction yield, and therefore used for examining various biological activities including anti-viral and anti-oxidant activity of different plant extracts as reported earlier [22–25]. In this study, the 50 g of dried leaves powder of both plants was dissolved in 250 ml of 100% methanol, followed by incubation for 24 h at 150 rpm and 25 °C on a rotary shaker. After incubation, the mixture was allowed to stand for 10 min for the sedimentation and the supernatant was filtered through Whatman filter paper no. 1. The extracted samples were collected and placed on a rotary evaporator at 60 °C for methanol evaporation. The leaves extracts were then collected by protecting them from direct light and kept at 4 °C for further characterization and phytochemical analysis through High-resolution electrospray ionization mass spectrometry (HR-ESI–MS).

### Phytochemical characterization and Identification of bioactive components from *C. papaya* and* P. guajava* leaves extract

Qualitative phytochemical tests were performed to check the presence of carbohydrates, flavonoids, saponins, tannins, terpenoids, cardiac glycosides, steroids, quinones and coumarins using standard protocols as described earlier by Shaikh and Patil, 2020 [26]. The high throughput HR-ESI–MS technique was used in this study to identify the chemical constituent and bioactive compounds present in the leaves extract of *C. papaya* and *P. guajava*. Briefly, 1 mg extract was dissolved in 1.5 ml LC–MS grade methanol and clarified through a 0.2 μm filter membrane. Further 10μ/L was injected into the HR-MS column. The HR-ESI–MS analysis was carried out on an Agilent 6530 Q-TOF (Agilent, USA) mass spectrometer connected to an HPLC Prime Infinity II 1260 system (800 bar). A dual electrospray ionization (ESI) source was used for the ionization process. For LC-based metabolite separation, a Hypersil GOLD C18 (2.1 × 150 mm, 1.9 μm particle size, Thermo Scientific, USA) column was used at 40 °C with a flow rate of 0.3 ml/min. Silica gel (60 − 100 mesh and 100 − 200 mesh, Hi-media, India) was used for the chromatography column.

### Cell lines and HIV-1 stock

TZM-bl cells (HeLa modified cell line; initially called JC53-bl; clone 13) were procured from the National Institute of Health (NIH)—HIV Reagent Program, and maintained in DMEM (Gibco, USA) containing 10% FBS (Moregate, Australia) and supplemented with HEPES (Gibco, USA), antibiotics (Sigma, USA) at 37 °C in a 5% CO_2_ humidified chamber.

The primary isolates of HIV-1_UG070_ (X4, Subtype D) and HIV-1_VB028_ (R5, Subtype C) were obtained from the virus bank repository maintained at the Division of Virology, ICMR-National AIDS Research Institute, Pune.

### Cytotoxicity assay by MTT

Cytotoxic effect of both the plant extract *C. papaya* and *P. guajava* was performed in the TZM-bl cell line following the methods described earlier [27, 28]. Briefly, 1 × 10^5^ adherent TZM-bl cells/well were seeded on 96 well plate and incubated for 24 h supplied with 5% CO_2_ at 37 °C. The purified phyto-extract dilutions were treated on the cell-seeded plates in dose dependent manner by taking an initial concentration of 6 mg/ml for *C. papaya* and 8 mg/ml for *P. guajava*, and incubated for 48 h. After the incubation period, the phyto-extracts were evaluated by adding 20 µl (5 mg/ml) MTT to all wells and further incubated for 3 h, which allows the MTT to get metabolized; the supernatant was replaced with 150 µl dimethyl sulfoxide (DMSO) to dissolve the formazan crystals. After the final incubation of an hour, the O.D. value was recorded at 550 nm and 630 nm using a multimode plate reader. The viability was observed based on a comparison with the absorbance of untreated and treated cells. The mean absorbance (O.D.) of duplicate wells was used to calculate the percentage of cell viability as follows: Percentage of cell viability = (Absorbance of Extract Treated Cells – Absorbance of Blank) / (Absorbance of Control – Absorbance of Blank) × 100%. The CC_50_ was obtained at the concentration where 50% of the cells remain viable in presence of the phyto-extracts from three independent assays.

### Cell associated assay (CA)

Based on the CC_50_ value, a range of non-cytotoxic concentrations of the *C. papaya* and *P. guajava* extracts were used, and the anti-HIV-1 activity was evaluated as described previously [28–31]. Briefly, the TZM-bl cells (1 × 10^4^ cells/well) were first infected with the virus HIV-1_VB028_ and HIV-1_UG070_ for 2 h at 37 °C in 5% CO_2_ incubator followed by the treatment with different dilutions of the extracts, along with the addition and incubation of 25 µg/ml DEAE-dextran for viral internalization. After 48 h post incubation, the luciferase activity was measured using the Britelite™ plus reagent on a luminometer (Perkin Elmer, USA) [31, 32]. Standard nucleoside reverse transcriptase inhibitor drug Azidothymidine or AZT was used at the known concentration of 0.45 µM/ml for both the phyto-extracts, as a positive control.

### Cell free assay (CF)

While in cell free assay (CF), the viral stocks were first treated with the serial dilutions of the *C. papaya* and *P. guajava* methanolic extracts and incubated for 1 h, at 37 °C in 5% CO_2_ atmosphere preceding its addition on the TZM-bl cells (1 × 10^4^ cells/well) [28–30]. At 48 h post-infection, the luciferase activity was measured as described above. Dextran sulphate (DS) was used as a positive control for the cell-free assays at the known concentration of 15 µg/ml for both the phyto-extracts.

The percentage of HIV-1 inhibition and EC_50_ value for both CA and CF assays were calculated based on the activity of the respective phyto-extracts. The results were compared with the positive controls after carrying out all the experiments in triplicate.

### Time-of-addition assay (TOA)

The Time-of-addition assay or TOA test was carried out as previously described with some modifications [33]. The designed assay, which included the positive and negative controls, was quite similar to that utilized for measuring the inhibitory potencies through anti-HIV-1 assays. In 96-well plates, TZM-bl cells (1 × 10^4^ cells/well) were seeded and after overnight incubation, the cells were infected with the HIV-1_VB028_ strain at 400 TCID_50_/ml. The inhibitors were either added to the wells concurrently (0hpi) or at different hours of post-infection as indicated (0.25-24hpi). At 48hpi the luciferase activity was assessed as described previously. The well-known anti-retrovirals Dextran sulphate (DS: Viral adsorption to the host cell inhibitor—15 µg/ml), Azidothymidine (AZT: An Nucleotide Reverse Transcriptase Inhibitors or NRTI—0.9 µM), Raltegravir (RAL: Integrase inhibitor—0.48 µM), Ritonavir (RTV: Protease inhibitor—45 µM), along with the *C. papaya* (1.25 mg/ml) and *P. guajava* (0.085 mg/ml) methanolic extract were employed in the experiment.

### HIV-1 protease (PR) inhibition activity assay

The *C. papaya* and *P. guajava* extracts were tested for HIV-1 protease inhibitory activity using an HIV-1 PR inhibitor screening Fluorometric assay kit following the manufacturer's instructions (Abcam, Cambridge, UK). Briefly, each sample was incubated with the HIV-1 PR enzyme for 15 min at room temperature. The fluorescent substrate was then added, and the PerkinElmer EnSpire plate reader was used to measure the fluorescence (excitation/emission = 330/450 nm) in a kinetic mode for 120 min at 37 °C. The kit-supplied Inhibitor Control (IC) Pepstatin (1 mM) and known protease inhibitor RTV (45 µM) were used as the positive controls, whereas, DMSO (1%, v/v) and kit-supplied Enzyme Control (EC) were used as the vehicle and negative controls, respectively, to normalize the background noise.

### Assessment of antioxidant activity

TZM-bl cells were seeded in 60 mm dishes at a density of 1 × 10^5^ cells per plate and placed in an incubator for 24 h at 37 °C with 5% CO_2_. After incubation, the cells were infected with HIV-1_VB028_ and were treated with the respective phyto-extracts having concentration determined by their CC_50_ and EC_50_ values. At 24hpi, the cells were trypsinized, collected and centrifuged at 2000 rpm for 5 min and incubated with 5 µM DCF-DA green molecular probe at 37 °C incubator. At 30 min post-incubation, the cells were centrifuged again, washed and resuspended in PBS, followed by filtration through nylon mesh, and the acquisition was done using FACS Aria flow cytometer (BD Bioscience, USA). Flow Jo™ software was utilized to analyze the data. A total of 50,000 cells from each sample set were examined during the assay.

### Free radical scavenging assay by DPPH method

DPPH (2,2-diphenyl-1-picryl-hydrazyl-hydrate) free radical method is an antioxidant assay based on electron transfer that produces a violet solution in ethanol [34]. 50 µl of each phyto-extract (*C. papaya* and *P. guajava*), based on their respective EC_50_, was added to a 96-well plate in triplicates. 100 µl of 0.2 mM of freshly prepared methanolic DPPH solution was added to the extracts, mixed, vortexed, and incubated on the 96-well plate at room temperature in a dark environment for 30 min. The absorbance was measured at 517 nm. Ascorbic acid was used as a standard and DPPH and methanol were taken as control. The DPPH scavenging effects were measured using the following formula: DPPH scavenging effect (%) = (A0 – A1/A0) × 100; where, A0 = Absorbance of Control; A1 = Absorbance of Sample.

## Results

In this study, we aimed to characterize the bioactive compounds of *C. papaya* L and *P. guajava* methanolic leaves extract, and identified the presence of multiple phytoconstituents through the High-resolution electrospray ionization mass spectrometry analysis. Further, we evaluated the anti-HIV-1 and antioxidant activity of these two methanolic phyto-extracts against the HIV-1 primary isolates for anti-retroviral drug screening.

### Phytochemical screening and structural elucidations of isolated compounds

Qualitative phytochemical screening was done using standard phytochemical tests for different bioactive compounds of methanolic leaves extract of *C. papaya* and *P guajava*. In our phytochemical analysis, we found the absence of saponins and tannins in both phyto-extracts, but the presence of reducing carbohydrates, terpenoids, cardiac glycosides, steroids, coumarins and quinones. However, flavonoids are detected only in the papaya leaf extract (Table S1).

**Fig. 1:**
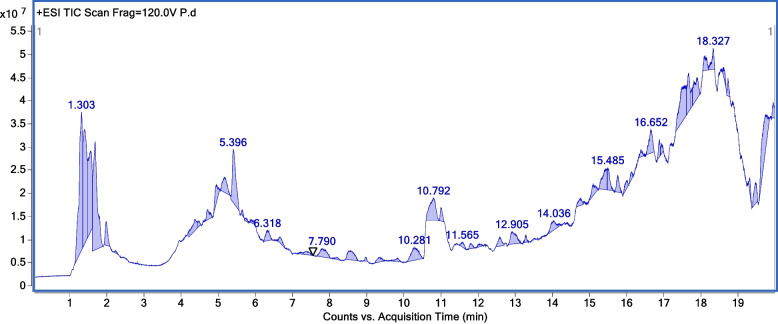
Chromatogram fragmentation of High-resolution electrospray ionization mass spectrometry analysis of *Carica papaya* Linn methanolic leaves extract

**Fig. 2:**
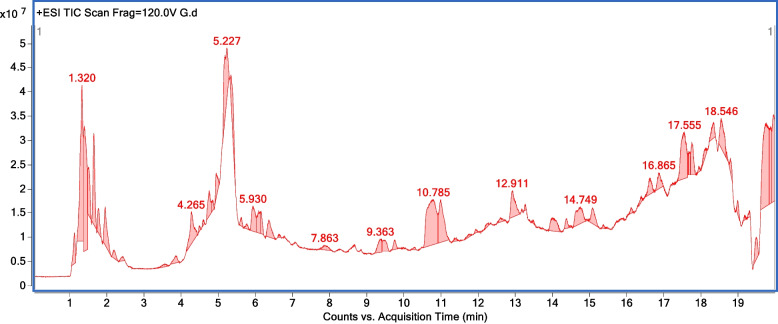
Chromatogram fragmentation of High-resolution electrospray ionization mass spectrometry analysis of *Psidium guajava* methanolic leaves extract

Further, the compounds were identified using the chromatogram fragmentation patterns and compared with highly advanced metabolites searched against the METLIN database (https://metlin.scripps.edu), filtered with a score > 80% in the computer library with their retention time for HR-ESI–MS extract of *C. papaya* and *P. guajava*, respectively (Figs. 1 and 2). The detailed list of identified compounds for *C. papaya* and *P. guajava* leaves extract are recorded in the supporting information (Table S2 and Table S3). The HR-ESI–MS fragmentation shows that both the phyto-extracts are enriched in various steroids, terpenoids, phenolic compounds, and fatty acids. The characterization of *C. papaya* leaves extract shows 25% of alkaloids (peptides, amino acids), 5% of glycoside, 10% lipids, 20% of phenolic compounds (aromatic phenol, quinone, flavonoids), 20% of terpenes, 15% of aliphatic compounds (fatty acids, alcohol, saturated and unsaturated alkenes) as well as 5% of other bioactive compounds (Table S2). Similarly, *P. guajava* extract shows 40% of alkaloids (peptide, amino acids), 10% of glycoside, 10% lipids, 10% of phenolic compounds (aromatic phenol, quinone), 10% of terpenes, 15% of aliphatic compounds (fatty acids, alcohol, saturated and unsaturated alkenes) and 5% of other compounds (Table S3). It was observed that both the phyto-extracts have a higher percentage of alkaloids terpenes.

### *In vitro* cytotoxicity of* C. papaya L* and* P. guajava* leaves extract on TZM-bl cells

**Fig. 3:**
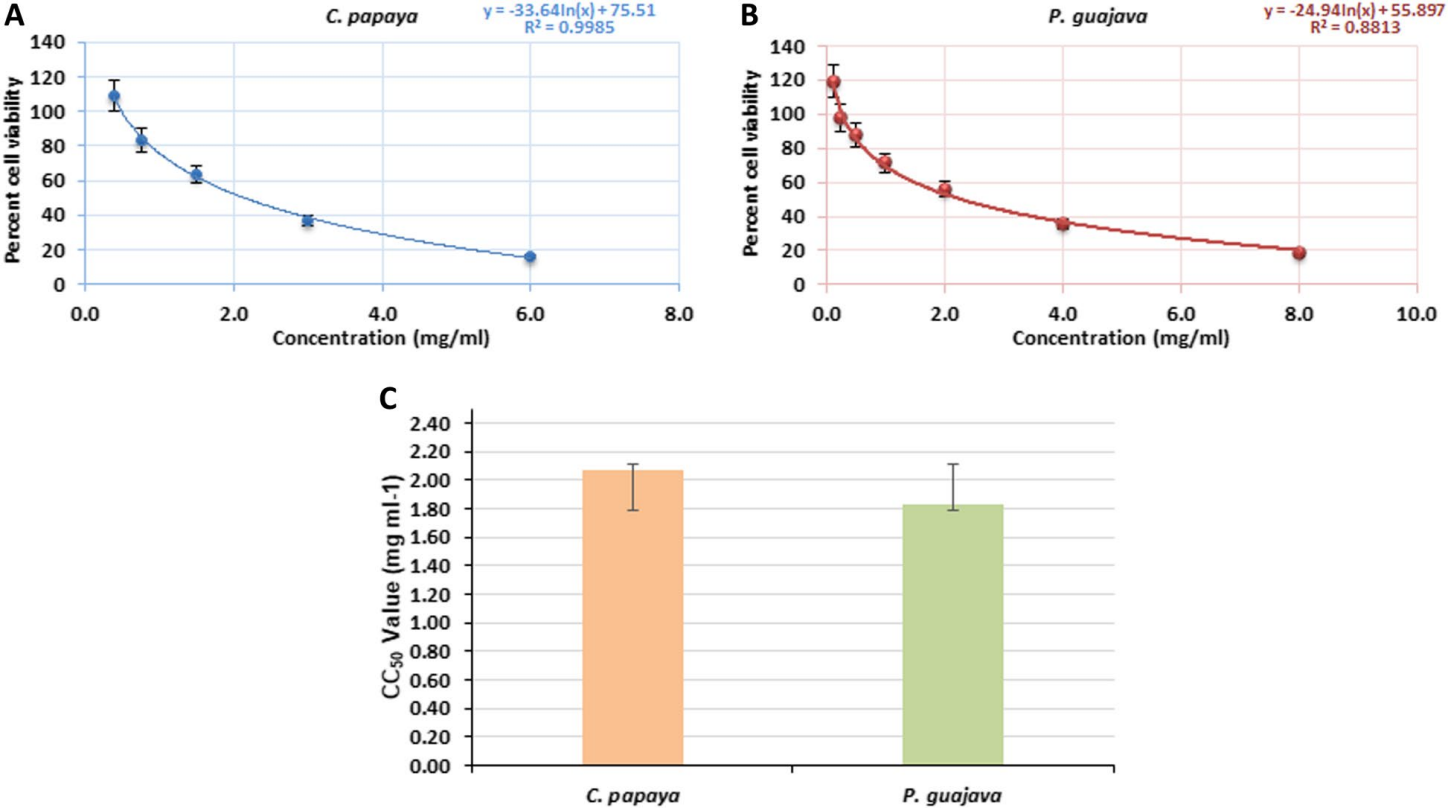
Determination of cytotoxic concentration of phyto-extracts. The effect of different concentrations of (**A**) *Carica papaya* Linn and (**B**) *Psidium guajava* extract on TZM-bl cell viability. **C** The comparative graphical illustration of CC_50_ concentration of *C. papaya* and *P. guajava*

Initially, the phyto-extracts of *C. papaya* and *P. guajava* were screened to assess their effects on the cellular viability of TZM-bl cells by MTT quantitative colorimetric assay. The dose-dependent effect of *C. papaya* (0.375–6.0 mg/mL) and *P. guajava* (0.125–8.0 mg/mL) on cell viability was represented for the concentrations of the phyto-extracts against the percentage of viable cells (Fig. 3A and B). The concentration that allows the 50% cells viable or the CC_50_ values for *C. papaya* and *P. guajava* were calculated to be 2.07 and 1.84 mg/ml, respectively, from three independent replicates (Fig. 3C).

### Anti-HIV-1 activity of *C. papaya* and *P. guajava* leaves extract

The TZM-bl cells were used for the screening of anti-HIV-1 activity. Based on the CC_50_ values, the concentration of 1.5 mg/ml was selected for both the phyto-extracts, as the percentage cell viability at this concentration is 69.68% and 65.17% for *C. papaya* and *P. guajava*, respectively. The ability of the *C. papaya* and *P. guajava* to inhibit its replication in the cell-associated (CA) and cell free (CF) virus was assessed using two different clades of HIV-1.

### Phyto-extracts mediated inhibition of HIV-1 replication

**Fig. 4:**
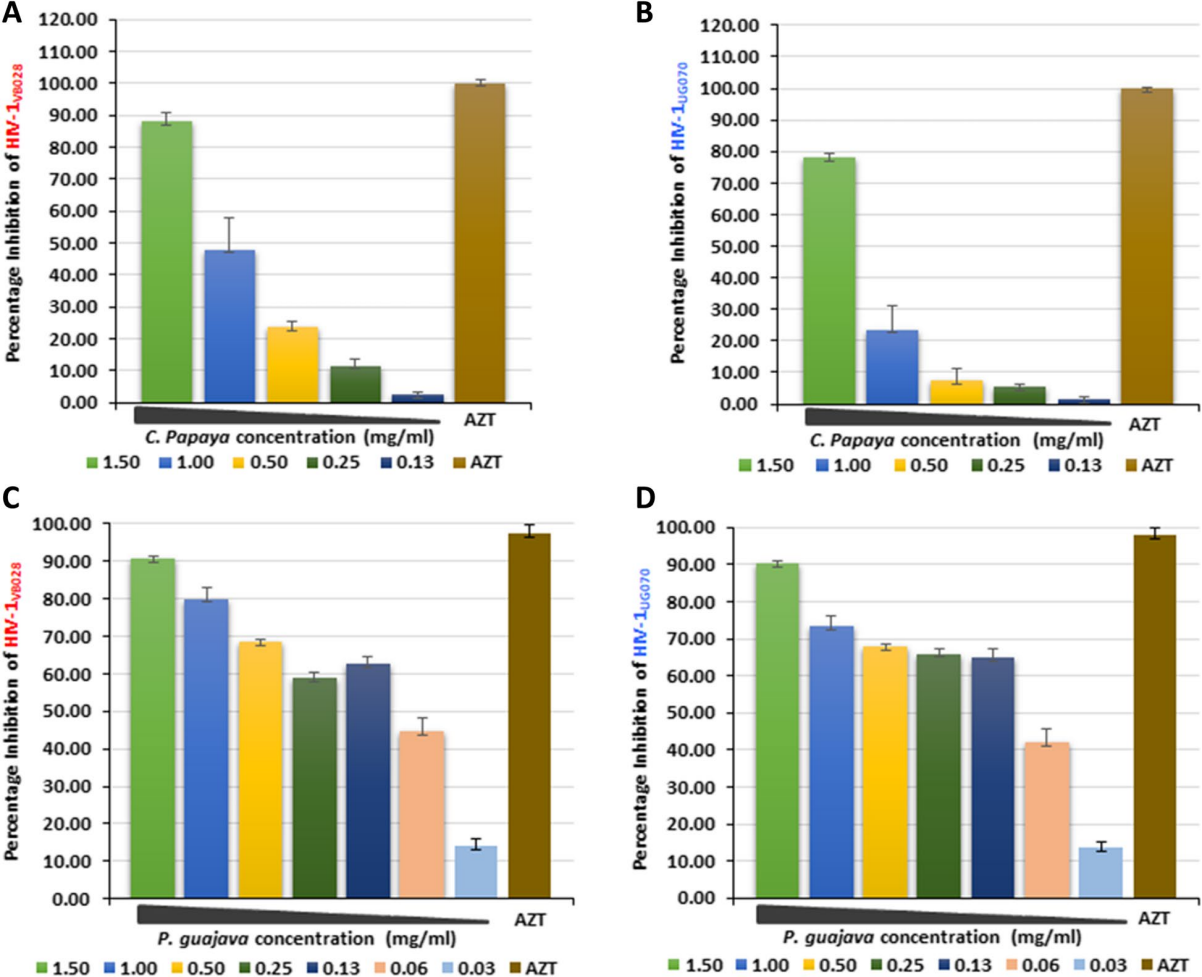
Anti-HIV-1 activity of *C. papaya* and *P. guajava* in cell associated study. Dose-dependent inhibition of (**A**) HIV-1_VB028_ and (**B**) HIV-1_UG070_ replication in presence of *C. papaya* extract (0.125–1.500 mg/ml). The effect of different concentrations of *P. guajava* extract (0.03125–1.500 mg/ml) on (**C**) HIV-1_VB028_ and (**D**) HIV-1_UG070_ replication. Standard drug Azidothymidine (AZT) at (0.45 µM/ml) was used as the positive control of HIV-1 inhibition

In the cell associated assay, the half maximal effective concentration EC_50_ values of the *C. papaya* extract against HIV-1_VB028_ (R5, Subtype C) and HIV-1_UG070_ (X4, subtype D) were 1.03 mg/ml and 1.25 mg/ml, whereas 0.070 mg/ml and 0.085 mg/ml for *P. guajava*, respectively. It was observed that the *C. papaya* and *P. guajava* showed a dose-dependent anti-HIV-1 activity in the TZM-bl cells (Fig. 4 and Figure S1). *C. papaya* extract exhibited significant inhibition of the replicating CA virus at the minimum concentration of 1–1.5 mg/ml (Fig. 4A and B; Figure S1A and B), whereas, *P. guajava* showed a consistent inhibition across the different concentrations (0.03125–1.5 mg/ml (Fig. 4C and D; Figure S1C and D).

### Suppression of HIV-1 transmission through cell-free assays

**Fig. 5:**
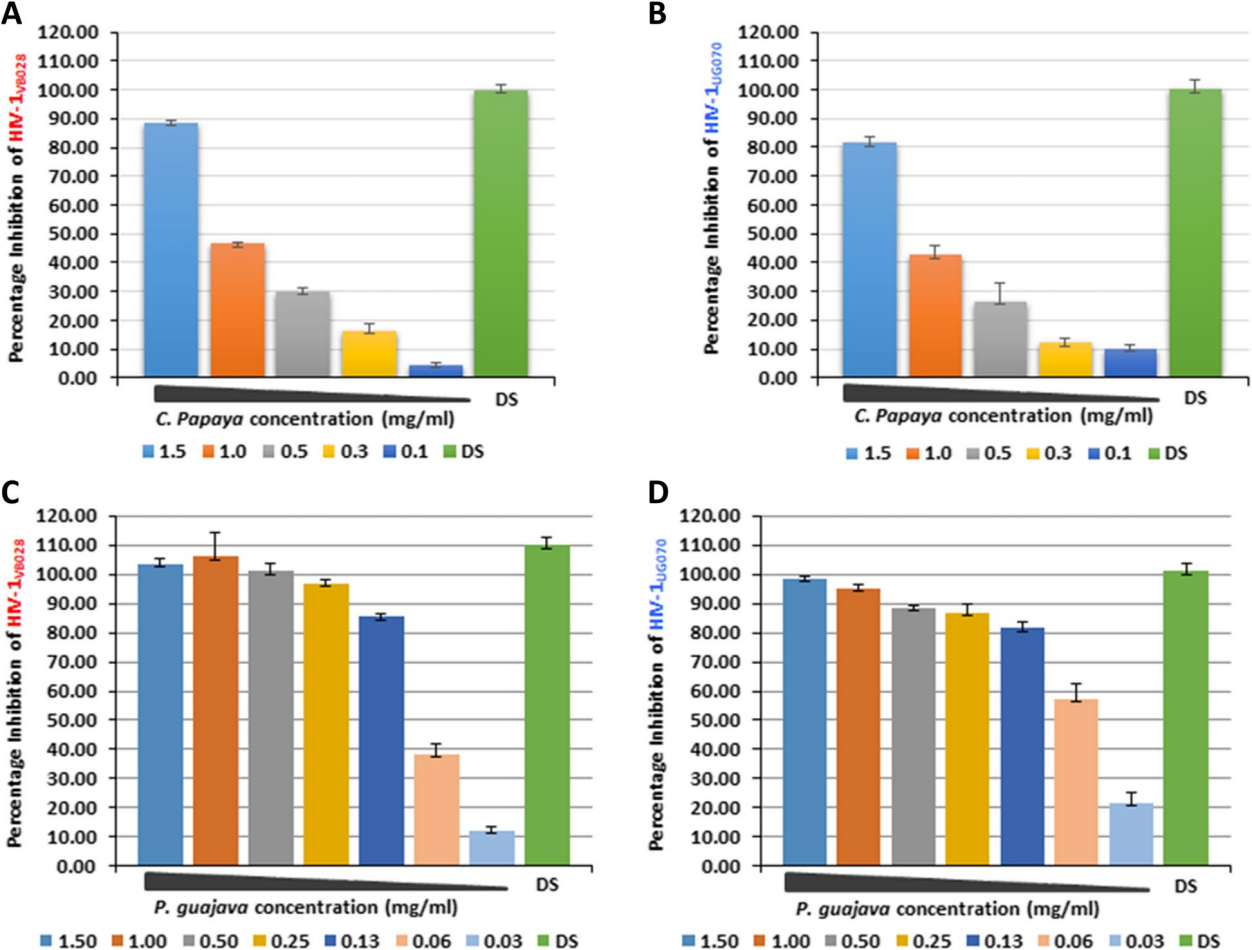
Effects of *C. papaya* and *P. guajava* on HIV-1 suppression through cell-free assays. Percentage of inhibition observed in dose dependent manner for (**A**) HIV-1_VB028_ and (**B**) HIV-1_UG070_ in presence of *C. papaya* extract (0.125-1.500 mg/ml). The effect of different concentrations of *P. guajava* extract (0.03125–1.500 mg/ml) on (**C**) HIV-1_VB028_ and (**D**) HIV-1_UG070_ isolates. The results of cell-free assays were compared with Dextran sulphate (DS) at 15 µg/ml as the positive control of HIV-1 inhibition

Apart from the viral replication within infected cells and the cell-to-cell transmission, the HIV-1 can also disseminate between CD4^+^ T lymphocytes by cell-free diffusion. Therefore, we examined the anti-HIV-1 potency of the *C. papaya* and *P. guajava* extracts in the cell-free system (Fig. 5 and Figure S2). The EC_50_ values of the *C. papaya* extract against HIV-1_VB028_ (R5, Subtype C) and HIV-1_UG070_ (X4, subtype D) were 1.075 mg/ml and 1.176 mg/ml whereas 0.073 mg/ml and 0.054 mg/ml for *P. guajava*, respectively. Likewise the cell associated assay, we observed significant inhibition of HIV-1 infection at the concentration of 1–1.5 mg/ml for *C. papaya* (Fig. 5A and B; Figure S2A and B) and 0.0625–1.5 mg/ml for *P. guajava* (Fig. 5C and D; Figure S2C and D) leaves extract in both X4 and R5 subtypes.

### Determining the phyto-extracts targets of action against HIV-1

**Fig. 6:**
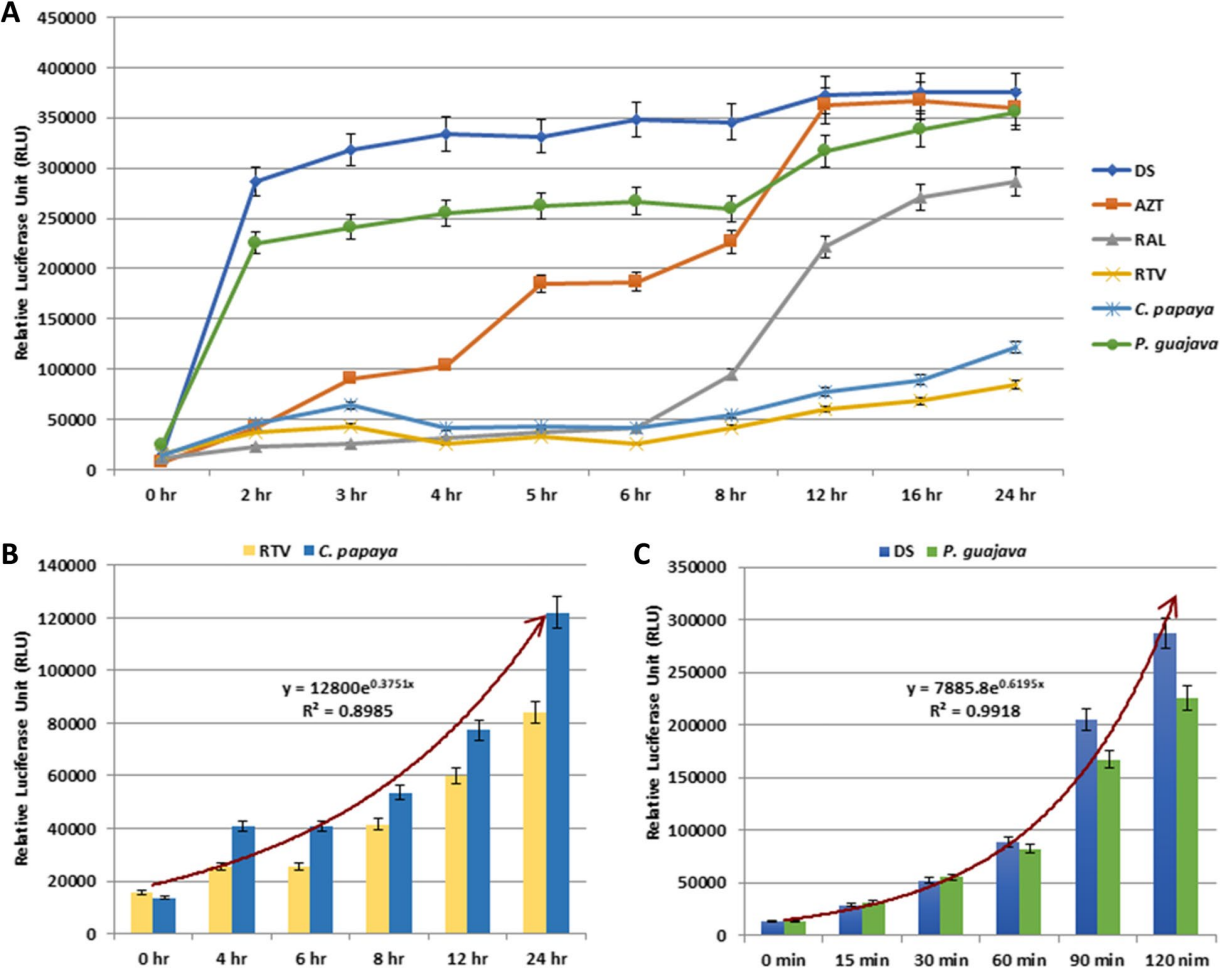
Mode-of-action of *C. papaya* and *P. guajava* through TOA assay. **A** The target of methanolic extract of *C. papaya* and *P. guajava* was compared to known antiretroviral drugs. Final concentrations of drugs and extracts were introduced at various time intervals concurrently and/or after HIV-1_VB028_ infection as indicated. Comparative analysis of loss-of-inhibition profile of (**B**) *C. papaya* and (**C**) *P. guajava* with known HIV-1 PR inhibitor (RTV) and HIV-1 entry inhibitor (DS), respectively. The loss-of-inhibition profile was calculated in terms of Relative Luciferase Unit (RLU). The data represented are the mean and standard deviation of at least three independent experimental replicates

To identify the possible targets of interaction of the *C. papaya* and *P. guajava* leaves extract, and to provide the basis for further investigations, the time-of-addition assay was conducted with the phyto-extracts and known antiretrovirals (Fig. 6). The resulting *C. papaya* extract profile shows the loss-of-inhibition at 16hpi, a profile similar to Ritonavir (RTV), which is a known protease inhibitor (Fig. 6A and B). Whereas, the loss-of-inhibition profile of *P. guajava* extract exhibited even earlier than it was observed for the viral entry and adsorption to the host cell inhibitor Dextran Sulphate (DS) (Fig. 6A and C). This TOA analysis indicates the inhibitory effect of *C. papaya* extract through the suppression of HIV-1 protease, while the mode-of-action of *P. guajava* extract by blocking the viral entry to the cell, overall inhibiting the HIV-1 infection.

**Fig. 7:**
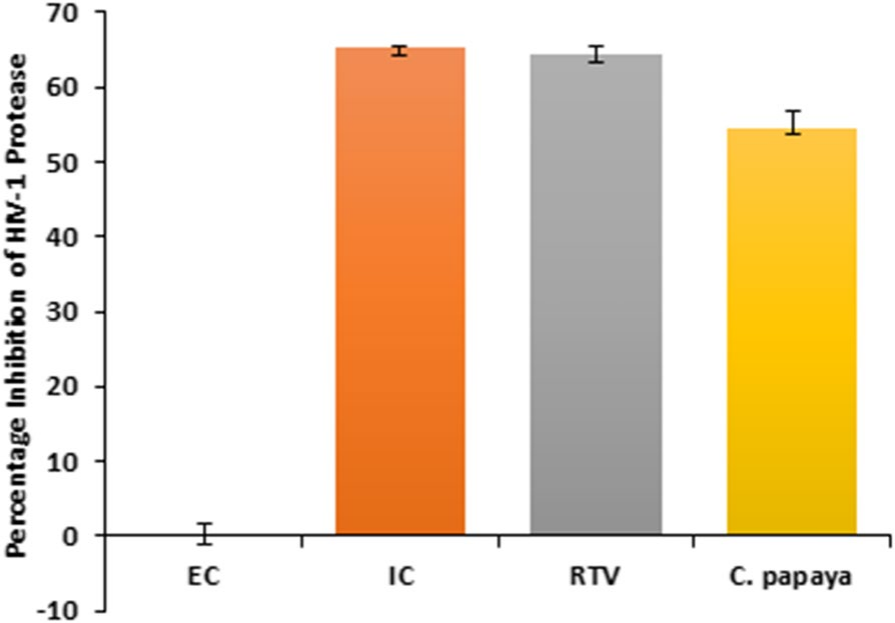
Protease Inhibition assay of *Carica papaya* Linn leaves extract. The percentage inhibition of HIV-1 protease enzyme activity in the presence of *C. papaya* (1.25 mg/ml) compared to the known HIV-1 PR inhibitor RTV (10 μM) and kit provided Inhibitor Control (IC), Pepestatin (1 mM). Enzyme Control (EC) represents the negative control to normalize the background fluorescence

Furthermore, we confirmed the protease activity of *C. papaya* extract against HIV-1 PR through the kit based in vitro HIV-1 protease inhibition assay. The result revealed 74.29% inhibition of HIV-1 protease activity at the given concentration of 1.25 mg/ml of *C. papaya* leaves extract (Fig. 7). The result was compared with known HIV-1 protease inhibitor Ritonavir (10 μM) as a positive control and the assay was validated with the kit provided Inhibitor control, Pepstatin (1 mM) and Enzyme control (EC).

### ROS scavenging activity of phyto-extracts in HIV-1 infected cells

**Fig. 8:**
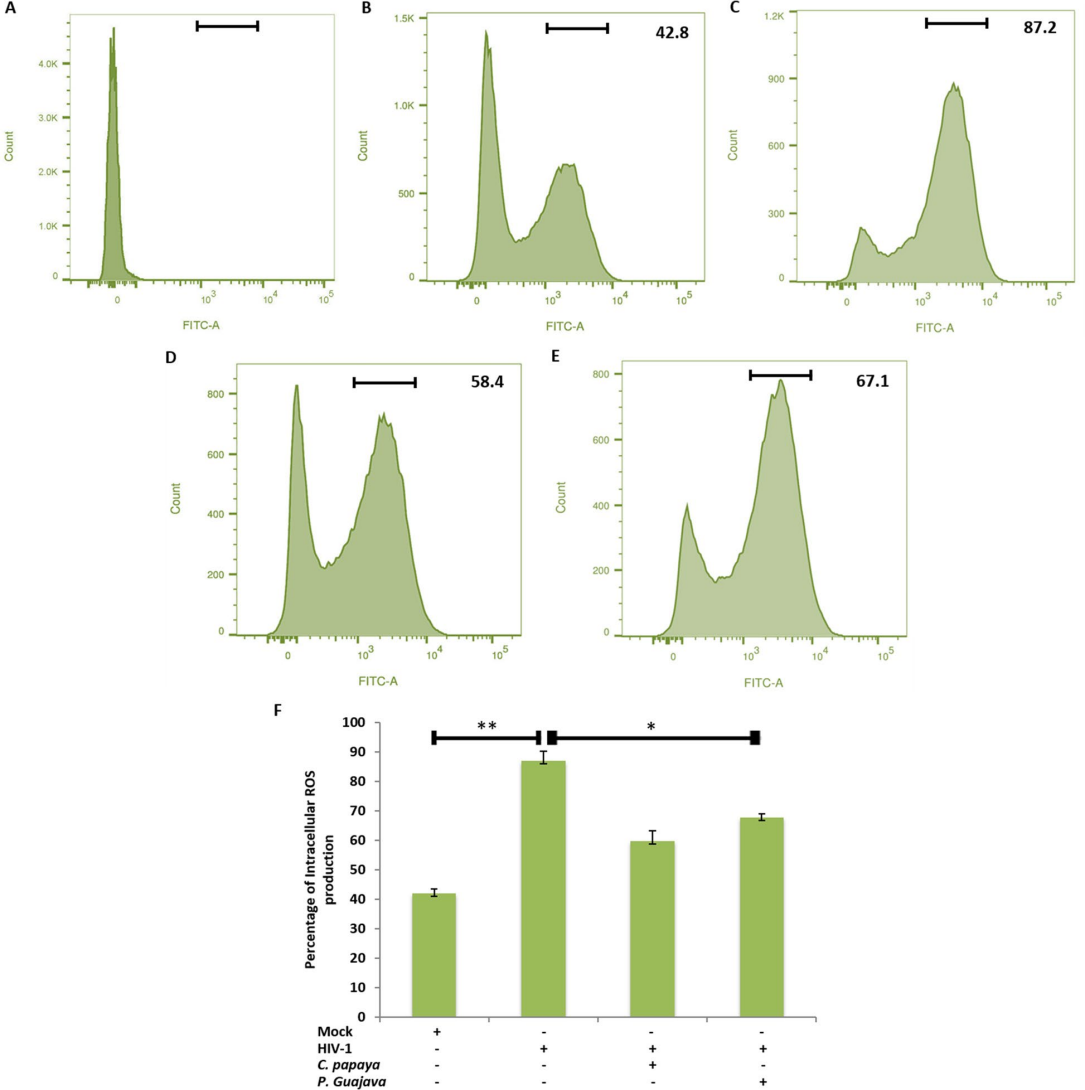
ROS Scavenging effects of *C. papaya* and *P. guajava* in HIV-1 infected cells. **A** Unstained untreated cells. **B** ROS generation in cell control (CC). **C** ROS generation in virus control (VC). **D** ROS inhibition in HIV-1 infected cells treated with *C. papaya* leaves extract. **E** Inhibition of intracellular ROS production in HIV-1 infected but *P. guajava* leaves extract treated cells. In each FACS image acquisition, fifty thousand cells were examined. The image group is representative of the three independent replicates. **F** Comparative analysis from the mean of three independent assays for intracellular ROS generation in HIV-1 infected and/or *C. papaya* and *P. guajava* leaves extract treated cells. * *p* < 0.05 and ** *p* < 0.01

Reactive oxygen species or ROS are short lived and highly reactive molecules, and high doses of ROS activate the cell death signaling pathways, i.e. apoptosis and necroptosis. During the pathological condition, ROS elevation was detected by using fluorescent-based molecular probe 2’,7’ Dichlorodihydroflurescin diacetate (DCFH_2_-DA). This dye is known to assess the activity of hydroxyl, peroxyl and other mitochondrial ROS within the cells. In the presence of hydrogen peroxide, DCFH_2_-DA oxidized into fluorescent Dichloroflurescin DCF while emits fluorescence, which was detected by FACS analyzer. The unstained untreated cells was used to nullify the background noise (Fig. 8A). The DCF fluorescence, observed under the normal homeostasis, in control cells was 42.8%, whereas, the virus control showed 87.2% (Fig. 8B and C). After 24 h post infection, cells showed increase in fluorescence accumulation as compared to the control cells. However, *C. papaya* treated cells resulted a noteworthy decrease in intracellular ROS production as evident with the DCF fluorescence value of 58.4% (Fig. 8D), while *P. guajava* treatment resulted decrease in fluorescence to 67.1% (Fig. 8E). The mean of three independent assays was analyzed statistically to examine the significance of ROS production in the cells treated with *C. papaya* and *P. guajava* extracts (Fig. 8F). An unrelated ROS generator (H_2_O_2_) and its scavenger (Catalase) were also used as additional controls (Figure S3). Together these results clearly showed that the phyto-extracts treatment eventually decreased the level of intracellular ROS in HIV-1 infected cells suggesting that the presence of *C. papaya* and *P. guajava* extracts ameliorated the production of ROS and having significant ROS scavenging potential.

The free radical scavenging activity of *C. papaya* and *P. guajava* leaves extract was further elucidated by DPPH assay, and was found to be 12.5% and 10.5% respectively (Table 1). Overall, *C. papaya* was found to have higher antioxidant capacity as compared to *P. guajava* extracts.

**Table 1:** Spectrophotometrically recorded DPPH scavenging activity of *Carica papaya* L and *Psidium guajava* leaf extracts

Plant Extracts	Absorbance (517 nm)	% DPPH scavenging activity
*Carica papaya* Linn	0.322 ± 0.040 ^a^	12.5%
*Psidium guajava*	0.33 ± 0.078 ^a^	10.3%

^a^ ± SD of 3 replicates

## Discussion

In this study, the biological activities of methanolic leaves extract from *Carica papaya* Linn and *Psidium guajava* were evaluated. The phytochemical profile of these two extracts were characterized by high throughput HR-ESI–MS analysis and revealed the presence of different bioactive constituents (Figs. 1 and 2; Table S2 and S3). The detailed in vitro studies indicated that both the leaves extract are less cytotoxic in nature (Fig. 3), with pronounced anti-HIV-1 activity (Figs. 4 and 5); while the TOA assay revealed the targets of action against HIV-1 (Fig. 6). In vitro enzymatic validation also confirmed the inhibitory role of *C. papaya* extract against HIV-1 protease (Fig. 7). Additionally, the ROS scavenging activity of these phyto-extracts unveiled their antioxidant potential during HIV-1 infection (Fig. 8 and Table 1). Overall, the results indicate synergistic action of bioconstituents of *C. papaya* and *P. guajava* leaves extract lead to antiviral and antioxidant activity in HIV-1 infected cells.

In absence of non-toxic antiretroviral drug therapies, lack of vaccine, diversity in viral strains, and emergence of resistant viruses often led the researchers for continuous search of alternative antivirals and new lead molecules to prevent the deleterious effect of HIV-1 infection. Investigation on bioactive molecules from natural plant sources has been one of the best strategies for the treatment of various infectious diseases, including HIV-1. Natural products are therefore regaining appeal in drug development as they circumvent the limitations of synthetic libraries, such as their lack of chemical variety [35]. Additionally, natural products have historically shown to be excellent starting points for the development of pharmaceuticals, with 34% of medications authorized by the FDA between 1981 and 2010 were from natural sources [13]. Numerous active ingredients of natural products and their derivatives, including alkaloids, quinones, flavonoids, terpenoids, glycans, organic acids, and others, have antiviral action [36]. According to a previous report, of all small-molecule drugs created in the last 28 years, 63.1% were natural product-based therapies [13]. This number indicates the enormous potential for new medicine discovery offered by natural product and their derivatives.

*C. papaya* and *P. guajava* represent an important source of phenolic compounds, some of which have been shown to have inhibitory effects against a number of viral infections [37, 38]. In particular, terpene compositions were found to be effective against SARS-CoV-2 and alkaloids isolated in *I. indigotica* roots were reported to reduce the influenza infection as well as the viral neuraminidase activities in vitro [39–41]. Furthermore, alkaloids, phenolic compounds, and terpenes are known bioactive components that have been shown to have antioxidant properties and to have an influence on oxidative stress and related signalling pathways [42–45].

In vitro viability testing has become an essential step in contemporary drug discovery, as it characterizes a compound's hazardous potential and gives proof of its safety index [46, 47]. Hence, crude methanolic extracts of the fruit plant *Carica papaya* Linn and *Psidium guajava* were tested for cytotoxicity in the TZM-bl cell line using MTT-based cell viability assay. The crude extracts were found to be less cytotoxic to the cells, as exhibited by the CC_50_ values, which were recorded as 2.07 and 1.84 mg/ml respectively for *C. papaya* and *P. guajava* in this study (Fig. 3 and Table 2), in accordance with the previous reports on other methanolic plant extracts those are already known to be low cytotoxic in nature [48]. Effects of *C. papaya* and *P. guajava* have already been documented with no detectable side effects that could be considered harmful, mutagenic, or otherwise aberrant [17–20].

**Table 2:** Summary of CC_50_, EC_50_ and SI of *Carica papaya* L and *Psidium guajava* leaf extracts

**Extract(s)**	**Cell Cytotoxicity (**^a^**CC50, mg/ml)**	**Anti-HIV-1 Activity Cell—Associated Assay**				**Anti-HIV-1 Activity Cell—Free Assay**			
		**HIV-1**_**VB028**_		**HIV-1**_**UG070**_		**HIV-1**_**VB028**_		**HIV-1**_**UG070**_	
		^b^**EC**_**50**_	^c^**S.I**	**EC**_**50**_	**S.I**	**EC**_**50**_	**S.I**	**EC**_**50**_	**S.I**
*C. papaya L*	2.07	1.03	2.009	1.25	1.66	1.075	1.926	1.176	1.760
*P. guajava*	1.84	0.07	26.28	0.085	21.65	0.073	25.21	0.054	34.07

^a^CC_50_: The cytotoxic concentration of the extracts that caused the reduction of viable cells by 50%

^b^EC_50_: The effective concentration of the extracts that resulted in 50% inhibition in HIV-1 infection

^c^S.I.: Selective Index is the ratio of cell cytotoxicity to its biological activity *i.e.* CC_50_/EC_50_

All data presented are averages of three independent experiments

Many studies have shown the potential of phyto-extracts against the HIV/AIDS. In a recent investigation it was observed that the methanolic extract of *Curcuma aeruginosa* Roxb. plant suppressed the HIV-1 PR [49]. Similar to this, another group of researchers reported the inhibition of HIV-1 encoded viral proteins by the bioactive from of *Alnus firma*'s methanol extract [50]. Literature has revealed the medicinal properties of phytotherapic plants like *Carica papaya* Linn and *Psidium guajava* for their antiviral and antioxidant activity [15]. In this brief study, we focused only on the anti-HIV-1 property and the antioxidant potential of *C. papaya* and *P. guajava* phyto-extract. The in vitro screening assays showed that both the extracts significantly inhibited the cell associated viral replication and cell free transmission of HIV-1 using two clinical isolates HIV-1_VB028_ and HIV-1_UG070_ from two different subtype of the virus (Figs. 4 and 5; Table 2). Additionally, Selectivity Index (SI) revealed that both the extracts mentioned above had variable activities. The *C. papaya* extract demonstrated anti-HIV-1 activity against two different HIV-1 strains with SI values of 2.0 and 1.66 in cell associated assays, while cell free assays revealed SI values of 1.92 and 1.76 respectively. It's interesting to note that the SI index of *P. guajava* revealed as 26.28 and 21.65 in cell associated assays and 25.21 to 34.07 in cells free assays (Table 2). These results suggest that the *P. guajava* extract might act as more effective anti-HIV-1 agent over *C. papaya* extract. Further we also carried out time-of-addition (TOA) experiment (Fig. 6). This TOA method determines how long a compound may be added to a cell culture without losing its antiviral properties. An antiviral compound's relative location in the time scale can be used to determine the target comparing to a reference drug. If the unknown drug's profile resembles with the existing known anti-HIV drug, it is highly likely that the unknown drug is targeting through the same route, or at the very least one that is active at the same time [51]. *P. guajava* inhibition profile resembles dextran sulphate, which is an known inhibitor of viral entry and/or adsorption to the host cells. Earlier, *Cistus incanus* extract was also demonstrated to inhibit a very early step in the HIV-1 replication cycle comparable to a fusion inhibitor [33, 52]. In our experimental set up, *P. guajava* showed pattern similar to Dextran Sulphate, while *C. papaya* showed loss-of-inhibition profile similar to the HIV-1 protease inhibitor Ritonavir. Studies have revealed that any one molecule might have dual target, i.e., targeting two or more distinct phases of viral lifecycle by one such inhibitor, however the last target that can be blocked by the inhibitor during the viral replication will always be revealed by this experiment [51]. As in this study, the resulting *P. guajava* profile shows loss-of-inhibition at time points as observed for the entry and/or adsorption inhibitor DS, whereas, the loss of inhibition pattern and enzymatic validation confirmed the inhibitory role of *C. papaya* extract against HIV-1 protease (Fig. 7).

The loss of immunological cells, especially CD4^+^ T_H_ cells, is the defining feature of HIV-1 infection. According to the earlier study, the HIV-1 envelope glycoproteins are the prime cause for declined CD4^+^ cells in the infected patients, which in turn trigger the accumulation of oxidative stress within the cells and leads towards the cell death [53]. In this study, the DCFH_2_-DA method was used to determine the HIV-1 induced ROS formation in the living cells, where the intensity of the fluorescence was associated with the measurement of generated ROS within the cells [54]. The phyto-extracts treatment of *C. papaya* and *P. guajava* in the HIV-1 infected cells reduced the virus mediated ROS production significantly (Fig. 8). The reduction of ROS accumulation within the cells signifies the therapeutic effectiveness of these extracts as potential agents for the treatment of HIV-1 infection.

Recent studies have highlighted the manipulation of oxidative stress and antioxidant-dependent pathways to facilitate the novel strategies for HIV cure through preclinical in vitro studies and clinical trials [31, 55–58]. There are ample evidences on improved status of HIV-1 infected patients with the treatment of antioxidants that enhances the glutathione levels while lowering the lipid peroxidation [58]. The redox alterations is one of the crucial factors of HIV-1 pathogenicity, such as neurotoxicity and dementia, exhaustion of CD4^+^/CD8^+^ T-cells, predisposition to lung infections, and certain side effects of the antiretroviral therapy [56]. Thus, anti-HIV-1 activity of any compound with antioxidant effects may offer a new strategy for prevention and treatment. Hence, maintaining the antioxidant level is an important parameter of HIV-1 patient management. According to the literature and the high throughput characterization carried out during this study revealed that both the plant extracts are the enriched source of antioxidants (Table S2 and Table S3), and nutraceutical value with multiple health benefits [14, 15, 59, 60]. Although the involvement of the entire phyto-complex cannot be ruled out and perhaps the major limitation of this present study, our data indicates that the prime bioactive components, such as phenolic compounds, alkaloids, and terpenes, might be the key regulator of the antiviral effects of the *C. papaya* and *P. guajava* extracts. Together, these substances have the potential to affect both the virion life cycle and the host cells’ defense mechanism, primarily by reversing the redox imbalance, which is necessary for the viral infection to establish. However, the role of individual phytoconstituents on HIV-1 inhibition remains to be elucidated, in-depth mechanistic study would be the isolation of these phytoconstituents to unveil the plausible modus operandi. Overall, based on this study, it can be stated that the *Carica papaya Linn* and *Psidium guajava* plant extracts have anti-viral activity against HIV-1 with the anti-oxidant potential, hence need to be explored further.

## Conclusions

This study has characterized the presence of several bioconstitutents of enriched antioxidant properties in the leaves extract of the fruit plant *Carica papaya* Linn and *Psidium guajava*, and demonstrated that *C. papaya* and *P. guajava* extracts exhibit a dose-dependent inhibition against both the primary isolates HIV-1_UG070_ and HIV-1_VB028_ in cell associated, as well as in cell free assays. Furthermore, these extracts exhibited the radical scavenging activity against the HIV-1 induced ROS production within the cells, which further extenuates the viral replication. Thus, the future prospective work of isolation of the identified bioactive compounds and investigating their impact on the activity of viral encoded proteins those are crucial to the HIV-1 life cycle will be explored for additional antiviral strategies.

## Supplementary Information

**Additional file 1:** **Table S1.** Phytochemical constituents of methanolic leaves extract of *Carica papaya *and *Psidium guajava*. **Table S2**. Identified Compound from *Carica papaya* extract using HR-ESI-MS.*Carica papaya *extract shows 25% of alkaloids (Peptide, Amino acids), 5% of Glycoside, 10% lipids, 20% of Phenolic compounds (Aromatic Phenol, Quinone, Flavonoids), and 20% of Terpenes, 15% of Aliphatic Compounds (Fatty acids, alcohol and saturated, Unsaturated Alkenes) as well as 5% of other. We observed that both extracts have a higher percentage of alkaloids, Terpenes. **Table S3.** Identified Compound from *Psidium guajava *extract using HR-ESI-MS. The HR-ESI-MS data of *Psidium guajava *extract shows 40% of alkaloids (Peptide, Amino acids), 10% of Glycoside, 10% lipids, 10% of Phenolic compound (Aromatic Phenol, Quinone), and 10% of Terpenes, 15% of aliphatic Compounds (Fatty acids, alcohol and saturated, Unsaturated Alkenes) as well as 5% of other compounds were observed. **Figure S1.** Anti-HIV-1 activity of *C. papaya *and *P. guajava *in cell associated study. Dose-dependent inhibition of (A) HIV-1VB028 and (B) HIV-1UG070 replication in presence of *C. papaya *extract (0.125-1.500mg/ml). The effect of different concentrations of *P. guajava *extract (0.03125-1.500mg/ml) on (C) HIV-1VB028 and (D) HIV-1UG070 replication*. ***Figure S2.** Effects of *C. papaya *and *P. guajava *on HIV-1 suppression through cell-free assays. Percentage of inhibition observed in dose dependent manner for (A) HIV-1VB028 and (B) HIV-1UG070 in presence of *C. papaya *extract (0.125-1.500mg/ml). The effect of different concentrations of *P. guajava *extract (0.03125-1.500mg/ml) on (C) HIV-1VB028 and (D) HIV-1UG070 isolates. **Figure S3.** (A) ROS generator 15μM H_2_O_2_ (H_2_O_2_ generator) for 6 h served as positive control. (B) 15μM H_2_O_2_ (H_2_O_2_ generator) and 250U Catalase served as scavenger/inhibitor of ROS generation in the experiment.

## Data Availability

The original contributions presented in the study are included in the article/supplementary material, further inquiries can be directed to the corresponding author/s.
