# Supplementary material for: Antiviral and ROS scavenging potential of Carica papaya Linn and Psidium guajava leaves extract against HIV-1 infection
Source: BMC Complement Med Ther. 2023 Mar 18;23:82. doi: 10.1186/s12906-023-03916-x (PMC10024014; doi:10.1186/s12906-023-03916-x)
Supplement: Supplementary file 1 — Additional file 1: Table S1. Phytochemical constituents of methanolic leaves extract of Carica papaya and Psidium guajava. Table S2. Identified Compound from Carica papaya extract using HR-ESI-MS.Carica papaya extract shows 25% of alkaloids (Peptide, Amino acids), 5% of Glycoside, 10% lipids, 20% of Phenolic compounds (Aromatic Phenol, Quinone, Flavonoids), and 20% of Terpenes, 15% of Aliphatic Compounds (Fatty acids, alcohol and saturated, Unsaturated Alkenes) as well as 5% of other. We observed that both extracts have a higher percentage of alkaloids, Terpenes. Table S3. Identified Compound from Psidium guajava extract using HR-ESI-MS. The HR-ESI-MS data of Psidium guajava extract shows 40% of alkaloids (Peptide, Amino acids), 10% of Glycoside, 10% lipids, 10% of Phenolic compound (Aromatic Phenol, Quinone), and 10% of Terpenes, 15% of aliphatic Compounds (Fatty acids, alcohol and saturated, Unsaturated Alkenes) as well as 5% of other compounds were observed. Figure S1. Anti-HIV-1 activity of C. papaya and P. guajava in cell associated study. Dose-dependent inhibition of (A) HIV-1VB028 and (B) HIV-1UG070 replication in presence of C. papaya extract (0.125-1.500mg/ml). The effect of different concentrations of P. guajava extract (0.03125-1.500mg/ml) on (C) HIV-1VB028 and (D) HIV-1UG070 replication. Figure S2. Effects of C. papaya and P. guajava on HIV-1 suppression through cell-free assays. Percentage of inhibition observed in dose dependent manner for (A) HIV-1VB028 and (B) HIV-1UG070 in presence of C. papaya extract (0.125-1.500mg/ml). The effect of different concentrations of P. guajava extract (0.03125-1.500mg/ml) on (C) HIV-1VB028 and (D) HIV-1UG070 isolates. Figure S3. (A) ROS generator 15μM H2O2 (H2O2 generator) for 6 h served as positive control. (B) 15μM H2O2 (H2O2 generator) and 250U Catalase served as scavenger/inhibitor of ROS generation in the experiment. [file 12906_2023_3916_MOESM1_ESM.pdf]

# Supplementary Material

# Antiviral and ROS Scavenging Potential of Carica papaya Linn and Psidium guajava Leaves Extract against HIV-1 Infection

**Pratiksha Jadaun[1], Prachibahen Shah[2,†], Harshithkumar R[1,†], Madhukar S. Said[3,†], Shubhangi P. Bhoite[3,†], Sowmya Bokuri[2], Selvan Ravindran[2], Neetu Mishra[2] and Anupam Mukherjee[1,*]**

[†] These authors contributed equally to this work and share second authorship.

* Correspondence:
Dr. Anupam Mukherjee
amukherjee@nariindia.org; mukherjee.a@icmr.gov.in

**Table S1. Phytochemical constituents of methanolic leaves extract of *Carica papaya* and *Psidium guajava***

| Phytochemicals | Name of the test | *Carica papaya* | *Psidium guajava* |
|---|---|---|---|
| **Carbohydrates** | Molisch test | + | + |
| **Flavanoids** | Alkaline reagent test | + | - |
| **Saponins** | Foam test | - | - |
| **Tannins** | Ferric chloride test | - | - |
| **Terpenoids** | Chloroform-$H_2SO_4$ method | + | + |
| **Cardiac glycosides** | Keller Kilani test | + | + |
| **Steroids** | Salkowki test | + | + |
| **Coumarins** | Alkaline test | + | + |
| **Quinones** | $H_2SO_4$ method | + | + |

**(+) Shows positive and (-) negative results.**

**Table S2: Identified Compound from *Carica papaya* extract using HR-ESI-MS.** *Carica papaya* extract shows 25% of alkaloids (Peptide, Amino acids), 5% of Glycoside, 10% lipids, 20% of Phenolic compounds (Aromatic Phenol, Quinone, Flavonoids), and 20% of Terpenes, 15% of Aliphatic Compounds (Fatty acids, alcohol and saturated, Unsaturated Alkenes) as well as 5% of other. We observed that both extracts have a higher percentage of alkaloids, Terpenes.

| Formula | Mass | RT | Height | Area | Score | Name |
|---|---|---|---|---|---|---|
| $C_{54}H_{101}NO_8P$ | 922.7262 | 13.853 | 5118 | 25518 | 91.33 | PC(22:4(7Z,10Z,13Z,16Z)/24:0) |
| $C_{38}H_{75}NO_8P$ | 704.5241 | 17.422 | 24137 | 96926 | 90.42 | PC(14:0/16:1(9Z)) |
| $C_{36}H_{73}NO_8P$ | 678.5067 | 5.635 | 3083596 | 17150362 | 90.85 | PC(14:0/14:0) |
| $CH_5N_3$ | 59.0482 | 1.051 | 10433 | 20987 | 85.33 | Guanidine |
| $C_{10}H_{16}N_2O_8$ | 292.0902 | 7.25 | 49721 | 849686 | 86.85 | Edetate |
| $C_{10}H_{12}N_2O_3$ | 208.0853 | 5.97 | 34521 | 259885 | 89.44 | Formyl-5-hydroxykynurenamine |
| $C_{41}H_{78}NO_8P$ | 743.5457 | 15.717 | 43806 | 217552 | 85.44 | PE(18:2(9Z,12Z)/18:0) |
| $C_{40}H_{78}NO_8P$ | 731.5484 | 18.337 | 38288 | 174844 | 86.37 | PE(20:1(11Z)/15:0) |
| $C_{40}H_{72}NO_8P$ | 725.4994 | 14.649 | 20248 | 49069 | 85.23 | PE(20:4(5Z,8Z,11Z,14Z)/15:0) |
| $C_{39}H_{78}NO_8P$ | 719.5402 | 16.868 | 15159 | 21555 | 84.37 | PE(14:0/20:0) |
| $C_{10}H_{13}N_5O$ | 219.1125 | 18.214 | 36427 | 766036 | 85.27 | cis-Zeatin |
| $C_5H_9N_3$ | 111.0795 | 1.253 | 101510 | 32024 | 83.95 | Betazole |
| $C_4H_8N_4O_4$ | 176.0548 | 16.215 | 8068 | 18897 | 86.12 | Allantoic acid |
| $C_2H_7NO_2S$ | 109.0196 | 5.117 | 14233 | 25483 | 96.28 | Hypotaurine |
| $C_{14}H_{25}NO_{11}$ | 383.1414 | 6.112 | 20482 | 40775 | 91.68 | Beta-1,4-mannose-N-acetylglucosamine |
| $C_7H_{11}NO_5$ | 189.0634 | 5.83 | 49522 | 207388 | 89.67 | AminoDHQ |
| $C_{18}H_{29}D_5O_2$ | 287.2879 | 11.89 | 23706 | 69201 | 91.08 | Oleic acid (d5) |
| $C_{16}H_{24}N_2O_4$ | 308.173 | 15.147 | 36019 | 484368 | 94.25 | Bestatin |
| $C_{21}H_{34}O$ | 302.2588 | 11.87 | 25076 | 45717 | 97.34 | (15:1)-Cardanol |
| $C_7H_8NO_2$ | 138.0567 | 1.383 | 650136 | 1689048 | 90.47 | Trigonelline |
| $C_{11}H_{12}N_2O_3$ | 220.0846 | 15.236 | 7163 | 18959 | 86.24 | N-Hydroxy-L-tryptophan |
| $C_5H_{13}O_8P$ | 232.0346 | 1.575 | 217384 | 1116976 | 91.87 | D-Ribitol 5-phosphate |
| $C_{23}H_{30}N_2O_5$ | 414.2131 | 12.165 | 618110 | 5092427 | 85.44 | Desacetylvindoline |
| $C_8H_{19}N_6O_7P$ | 342.107 | 16.148 | 19380 | 96868 | 81.17 | Streptidine 6-phosphate |
| $C_{12}H_{21}NO_4S_2$ | 307.09 | 3.774 | 56692 | 426609 | 85.2 | S-Succinyldihydrolipoamide |
| $C_4H_8O_3$ | 104.0482 | 1.097 | 90102 | 427813 | 85.52 | D(-)-β-hydroxy butyric acid |
| $C_{30}H_{52}O_7P_2$ | 586.3229 | 19.014 | 662649 | 1850028 | 86.72 | Presqualene diphosphate |
| $C_{31}H_{55}O_9P$ | 602.3587 | 19.498 | 369234 | 1821060 | 92.08 | dolichyl beta-D-glucosyl phosphate |
| $C_{63}H_{105}NO_{12}P_2$ | 1129.7118 | 12.58 | 9764 | 48529 | 96.83 | N-Acetyl-D-glucosaminyldiphosphoundecaprenol |
| $C_9H_8O$ | 132.0586 | 1.25 | 652170 | 709649 | 93.05 | Atropaldehyde |
| $C_{21}H_{38}N_4O_8$ | 474.2691 | 17.03 | 510494 | 3495956 | 91.17 | Amastatin |
| $C_6H_{15}N_4O_6P$ | 270.0726 | 7.638 | 60066 | 573842 | 86.82 | D-Lombricine |
| $C_5H_{11}N$ | 85.0896 | 1.791 | 550113 | 630076 | 91.13 | Piperidine |
| $C_{30}H_{47}N_3O_9S$ | 625.3143 | 16.171 | 43403 | 252787 | 85.46 | Leukotriene C4 |

| | | | | | | |
|---|---|---|---|---|---|---|
| $C_{20}H_{40}O$ | 296.3121 | 15.014 | 18947 | 42611 | 92.36 | Phytol |
| $C_{25}H_{27}N_4O$ | 399.2168 | 17.216 | 168809 | 1984843 | 91.11 | Z-Phe-Phe-CHN2 |
| $C_6H_5NO_4$ | 155.0216 | 16.496 | 6682 | 14307 | 87.56 | 2,4-Dihydroxy-nitrophenol |
| $C_6H_{13}NO_2$ | 131.0948 | 2.441 | 738395 | 7496636 | 87.12 | L-Norleucine |
| $C_{18}H_{22}O_5S$ | 350.1194 | 17.846 | 17078 | 118070 | 88.28 | Estrone 3-sulfate |
| $C_8H_7NS$ | 149.03 | 19.216 | 10936 | 25357 | 91.91 | 2-Methylbenzothiazole |
| $C_6H_{12}O_6$ | 180.0654 | 1.515 | 2073667 | 14057729 | 85.83 | D-Tagatose |
| $C_{20}H_{20}N_2O_3$ | 336.1473 | 16.155 | 91052 | 959692 | 93.25 | Nb-Feruloyltryptamine |
| $C_4H_9N_3O_2$ | 131.07 | 4.706 | 27260 | 69423 | 87.51 | Creatine |
| $C_6H_{15}N_2O_6P$ | 242.0665 | 15.259 | 4623 | 14189 | 87.96 | 2-Deoxystreptamine 4-phosphate |
| $C_{26}H_{53}NO_7P$ | 522.363 | 19.123 | 2361701 | 23273042 | 84.91 | LysoPC(18:1(11Z)) |
| $C_7H_8O_8$ | 220.0231 | 1.257 | 641808 | 2174634 | 88.57 | 4-Carboxy-4-hydroxy-2-oxoadipate |
| $C_{18}H_{22}O_3$ | 286.1566 | 11.24 | 14705 | 54917 | 88.4 | Furamethrin |
| $C_4H_5N_3O_2$ | 127.0387 | 1.074 | 64087 | 299394 | 94.29 | I-Methyl-4-nitro-imidazole |
| $C_{14}H_{18}N_2O_4$ | 278.1268 | 14.036 | 25368 | 250598 | 96.89 | Prolyl-Tyrosine |
| $C_{12}H_{12}N_2O_4$ | 248.0812 | 17.17 | 46386 | 319594 | 90.78 | 5-Hydroxyindoleacetylglycine |
| $C_9H_{13}NO_6$ | 231.0748 | 5.973 | 37806 | 218952 | 87.5 | N-Acetyl-L-2-amino-6-oxopimelate |
| $C_5H_9NO_4$ | 147.0546 | 5.137 | 30042 | 118981 | 88.26 | O-Acetyl-L-serine |
| $C_{20}H_{14}NO_4$ | 332.094 | 2.003 | 179742 | 1002099 | 86.14 | SANGUINARINE |
| $C_{21}H_{29}FO_5$ | 380.1928 | 5.014 | 131003 | 927867 | 92.33 | Fludrocortisone |
| $C_{19}H_{25}N_5O_4$ | 387.1888 | 11.359 | 291256 | 2290450 | 91.71 | Terazosin |
| $C_7H_8N_4O_2$ | 180.0654 | 1.515 | 1910783 | 12571886 | 88.83 | Paraxanthine |
| $C_{15}H_{25}NO_3$ | 267.1839 | 4.713 | 23798 | 42883 | 87.29 | Metoprolol |
| $C_{31}H_{33}N_3O_6S$ | 575.2095 | 1.963 | 49640 | 185737 | 85.12 | Zafirlukast |
| $C_{13}H_{17}N$ | 187.1363 | 4.467 | 49475 | 347892 | 88.83 | (+/-)-2-(4'-Isobutylphenyl)propionitrile |
| $C_{26}H_{29}N_3O_6$ | 479.208 | 19.704 | 67070 | 772269 | 85.77 | Nicardipine |
| $C_8H_7N_3O_5$ | 225.0382 | 14.394 | 10851 | 83045 | 86.99 | Dinitolmide |
| $C_6H_{14}NO_2$ | 132.1027 | 2.441 | 423197 | 3332548 | 93.79 | beta-Alaninebetaine |
| $C_{22}H_{42}O_2$ | 338.3192 | 17.024 | 48028 | 215179 | 91.45 | cis-Cetoleic acid |
| $C_{14}H_{17}NO_{10}$ | 359.0866 | 10.596 | 97016 | 1310489 | 90.63 | TRIBOA-glucoside |
| $C_{17}H_{11}NO_7$ | 341.0521 | 10.633 | 75539 | 997278 | 93.19 | Aristolochic Acid |
| $C_{11}H_{13}N_3O_6$ | 283.0823 | 14.036 | 25847 | 259607 | 87.95 | Cucumopine |
| $C_{32}H_{46}N_2O_8$ | 586.3254 | 18.855 | 395562 | 2550746 | 87.51 | Inuline |
| $C_{24}H_{39}NO_7$ | 453.2737 | 19.475 | 69955 | 374036 | 88.66 | Delcosine |
| $C_{32}H_{44}N_2O_8$ | 584.3122 | 18.695 | 11774919 | 81571602 | 86.75 | Lappaconitine |
| $C_{33}H_{42}N_4$ | 494.3313 | 11.571 | 26792 | 107536 | 91.22 | Auricularine |
| $C_{38}H_{40}N_4O_2$ | 584.3218 | 18.957 | 329092 | 257280 | 95.98 | Caracurine V |
| $C_{43}H_{52}N_4O_6$ | 720.3896 | 17.107 | 59036 | 360925 | 92.52 | Epivoacorine |
| $C_{17}H_{22}O_6$ | 322.14 | 19.153 | 12819 | 59200 | 89.11 | Tetraneurin A |
| $C_{21}H_{21}NO_7$ | 399.1322 | 3.432 | 12715 | 159803 | 90.3 | Adlumidiceine |

| | | | | | | |
|---|---|---|---|---|---|---|
| **$C_{28}H_{36}N_2O_4$** | 464.2669 | 17.163 | 17638 | 11790 | 90.02 | Ipecac (Psychotrine) |
| **$C_{18}H_{22}O_{11}$** | 414.1167 | 7.565 | 17408 | 106656 | 86.06 | Asperuloside |
| **$C_{11}H_{13}NO_3$** | 207.089 | 1.704 | 596508 | 2145255 | 86.9 | Cantleyine |
| **$C_{39}H_{49}N_5O_7$** | 699.3676 | 13.157 | 66131 | 57463 | 94.01 | Lasiodine A |
| **$C_{24}H_{26}O_6$** | 410.1725 | 8.039 | 60279 | 572023 | 86.67 | α-Mangostin |
| **$C_8H_{15}NO_2$** | 157.1121 | 11.949 | 14981 | 47673 | 86.8 | Tranexamic acid |
| **$C_{41}H_{28}O_{26}$** | 936.0906 | 4.225 | 93182 | 307822 | 96.79 | Casuarinin |
| **$C_{31}H_{45}NO_8$** | 559.313 | 19.17 | 138319 | 476544 | 91.39 | Auriculine |
| **$C_{20}H_{27}NO_7$** | 393.1768 | 5.708 | 64573 | 541061 | 85.12 | Latifoline |
| **$C_{16}H_{11}NO_3$** | 265.0744 | 12.324 | 16053 | 100789 | 91.45 | Piperolactam A |
| **$C_{45}H_{73}NO_{14}$** | 851.4994 | 12.941 | 13053 | 24540 | 85.77 | alpha-Chaconine |
| **$C_{27}H_{48}N_2O$** | 416.378 | 16.778 | 93673 | 950044 | 88.11 | Cyclovirobuxine C |
| **$C_{27}H_{48}N_2O_2$** | 432.3714 | 15.77 | 239536 | 429354 | 94.97 | Solacapine |
| **$C_{22}H_{24}O_6$** | 384.1566 | 18.41 | 6822 | 17897 | 91.4 | 12-Dehydroporson |
| **$C_7H_7N_3$** | 133.0645 | 1.489 | 598515 | 1670793 | 86.54 | 3-Methylpyrrolo[1,2-a]pyrazine |
| **$C_{19}H_{17}NO_2$** | 291.1272 | 18.078 | 8267 | 29057 | 88.11 | Naproanilide |
| **$C_{13}H_7F_3N_2O_5$** | 328.0307 | 8.304 | 8037 | 53567 | 95.53 | Fluorodifen |
| **$C_{23}H_{36}N_4O_5$** | 448.267 | 17.657 | 24643 | 253155 | 90.83 | N-Acetyl-leu-leu-tyr-amide |
| **$C_{10}H_{15}O_4P$** | 230.0705 | 6.908 | 8638 | 23658 | 87.76 | Diethyl phenyl phosphate |
| **$C_{26}H_{44}O_9$** | 500.2982 | 13.495 | 217030 | 1101659 | 92.21 | Mupirocin |
| **$C_{26}H_{35}NO_4$** | 425.2568 | 7.124 | 76746 | 828491 | 88.61 | Diprenorphine |
| **$C_{41}H_{60}O_{11}$** | 728.4131 | 16.37 | 41283 | 153480 | 94.19 | Avermectin A1b monosaccharide |
| **$C_{26}H_{35}N_5O_{12}S$** | 641.1981 | 11.432 | 154630 | 1598893 | 87.48 | Cyclothialidine |
| **$C_{36}H_{48}N_2O_8$** | 636.3415 | 18.413 | 449009 | 3257077 | 92.9 | Ansatrienin A |
| **$C_{16}H_{14}N_2O_3$** | 282.1009 | 12.324 | 17490 | 105725 | 91.45 | Saphenic acid methyl ester |
| **$C_{16}H_{19}NO_4$** | 289.13 | 2.321 | 45572 | 1548787 | 92.04 | Balfourodine |
| **$C_{26}H_{33}NO_5$** | 439.2342 | 12.556 | 79780 | 768359 | 87 | Militarinone B |
| **$C_{30}H_{31}NO_9$** | 549.1988 | 9.532 | 22305 | 187050 | 87.29 | Jadomycin B |
| **$C_{19}H_{14}O_5$** | 322.0868 | 6.285 | 44577 | 318028 | 96.59 | 11-Deoxylandomycinone |
| **$C_{17}H_{14}N_2O_7$** | 358.0789 | 10.596 | 82135 | 1165905 | 94.84 | Coumeroic acid |
| **$C_{15}H_{22}FN_3O_6$** | 359.1494 | 1.28 | 154769 | 343989 | 93.09 | Capecitabine |
| **$C_{32}H_{32}O_{16}S_4$** | 800.0559 | 1.333 | 171790 | 624862 | 87.78 | TS-TM-calix(4)arene |
| **$C_{32}H_{61}NO_{10}P$** | 650.4025 | 17.359 | 122294 | 1671973 | 87.28 | 1-Palmitoyl-2-(5-hydroxy-8-oxo-6-octenoyl)-sn-glycero-3-phosphatidylcholine |
| **$C_{12}H_{11}N_3$** | 197.0945 | 1.721 | 117523 | 951786 | 86.64 | 2-Amino-3-methyl-9H-pyrido[2,3-b]indole |
| **$C_8H_6N_2O$** | 146.047 | 5.134 | 32262 | 199294 | 88.86 | 1(2H)-Phthalazinone |
| **$C_{15}H_{23}NOS$** | 265.1512 | 4.769 | 466119 | 2648362 | 86.86 | Esprocarb |
| **$C_{35}H_{43}NO_{11}$** | 653.2855 | 17.226 | 38938 | 162008 | 86.4 | Rifamycin W-hemiacetal |
| **$C_{20}H_{30}O_2$** | 302.2247 | 11.233 | 46768 | 588657 | 85.78 | Cascarillone |
| **$C_{22}H_{30}F_2O_3$** | 380.216 | 11.306 | 58711 | 614770 | 92.28 | 4,4-Difluoro-17beta-hydroxyandrost-5-en-3-one propionate |
| **$C_{13}H_{21}O_3PS$** | 288.0954 | 19.906 | 10737 | 38469 | 90.24 | Iprobenfos |

| | | | | | | |
|---|---|---|---|---|---|---|
| **$C_{14}H_{10}N_2O_4$** | 270.0636 | 17.17 | 46631 | 327290 | 93.51 | 4,4'-dinitrostilbene |
| **$C_4H_8FNO_3$** | 137.0493 | 1.383 | 658341 | 1730519 | 97.64 | 4-Fluoro-L-threonine |
| **$C_{10}H_{16}N_5O_9P$** | 381.069 | 10.629 | 5883 | 12340 | 86.51 | 2-Amino-5-formylamino-6-(5-phospho-D-ribosylamino)pyrimidin-4(3H)-one |
| **$C_{39}H_{56}N_6O_8$** | 736.4164 | 17.654 | 33197 | 200941 | 87.93 | Myxochromide S2 |
| **$C_{25}H_{45}NO_9$** | 503.309 | 18.629 | 67677 | 340461 | 85.06 | Pederin |
| **$C_7H_{17}N_3$** | 143.1432 | 17.77 | 9370 | 12333 | 86.57 | Dehydrospermidine |
| **$C_{30}H_{42}$** | 402.3311 | 9.999 | 19428 | 85537 | 85.14 | 4,4'-Diaponeurosporene |
| **$C_5H_8$** | 68.063 | 1.791 | 550074 | 630027 | 91.13 | Isoprene |
| **$C_{25}H_{27}NO$** | 357.2104 | 5.038 | 85480 | 513344 | 90.72 | N-Desmethyltamoxifen |
| **$C_{14}H_{22}N_2O_2$** | 250.1673 | 3.728 | 30199 | 207053 | 87.83 | Rivastigmine |
| **$C_{23}H_{27}NO_9$** | 461.1682 | 8.285 | 20146 | 197687 | 85.29 | Morphine 3-glucuronide |
| **$C_7H_{16}NO_4$** | 178.1072 | 1.326 | 22311 | 21505 | 86.98 | Anthopleurine |
| **$C_{25}H_{43}NO_{10}$** | 517.2878 | 17.183 | 2915546 | 47362111 | 88 | Mycalamide B |
| **$C_{26}H_{34}O_8$** | 474.2254 | 18.029 | 35581 | 127559 | 93.17 | Agrimophol |
| **$C_{26}H_{34}O_8$** | 474.2249 | 17.803 | 44549 | 346048 | 85.72 | Agrimophol |
| **$C_9H_{18}N_2O_2S$** | 218.1083 | 4.868 | 52158 | 165755 | 88.31 | Thiofanox |
| **$C_{18}H_{35}NO$** | 281.2726 | 17.455 | 158415 | 1727696 | 96.13 | Oleamide |
| **$C_{12}H_{19}N_3O_5$** | 285.1332 | 5.144 | 17730 | 29789 | 86.04 | Glycylprolylhydroxyproline |
| **$C_{24}H_{35}NO_5$** | 417.2507 | 12.201 | 95660 | 654263 | 89.33 | Batrachotoxinin A |
| **$C_{23}H_{28}O_8$** | 432.1771 | 8.092 | 45860 | 446413 | 90.97 | (2S,4S,6S)-2-[2-(4-Hydroxy-3-meyhoxyphenyl)ethyl]tetrahydro-6-(4,5-dihydroxy-3-methoxyphenyl)-2H-pyran-4-yl 4-acetate |
| **$C_{22}H_{31}NO$** | 325.2416 | 11.598 | 17206 | 107463 | 87.29 | Tolterodine |
| **$C_{18}H_{36}O_3$** | 300.2669 | 12.735 | 25843 | 104107 | 88.14 | (R)-10-hydroxystearic acid |
| **$C_{41}H_{28}O_{27}$** | 952.0994 | 4.109 | 968912 | 7530433 | 95.18 | Geraniin |
| **$C_{41}H_{30}O_{26}$** | 938.0987 | 4.354 | 102890 | 328235 | 85.09 | Eugeniin |
| **$C_{41}H_{26}O_{26}$** | 934.0718 | 3.926 | 1454647 | 10557650 | 90.28 | 2-O-Galloylpunicalin |
| **$C_{41}H_{64}O_{19}$** | 860.4062 | 12.327 | 93398 | 847262 | 91.93 | Corchorusoside E |
| **$C_{42}H_{74}O_{15}$** | 818.503 | 12.692 | 24968 | 156236 | 87.6 | Quinquenoside F1 |
| **$C_{41}H_{62}O_{15}$** | 794.4069 | 13.561 | 15198 | 41760 | 95.52 | Betavulgaroside IV |
| **$C_{34}H_{26}O_{22}$** | 786.0953 | 4.305 | 144479 | 865815 | 92.53 | Heterophylliin A |
| **$C_{42}H_{79}O_{10}P$** | 774.5383 | 19.879 | 108970 | 589250 | 92.83 | PG(18:0/18:2(9Z,12Z)) |
| **$C_{42}H_{61}NO_7$** | 691.445 | 15.316 | 79676 | 2848823 | 96.01 | 13-Demethylspirolide C |
| **$C_{45}H_{66}O_5$** | 686.4881 | 15.306 | 60953 | 799562 | 93.05 | DG(22:6(4Z,7Z,10Z,13Z,16Z,19Z)/20:5(5Z,8Z,11Z,14Z,17Z)/0:0) |
| **$C_{33}H_{60}O_{14}$** | 680.3922 | 12.825 | 609372 | 4726320 | 95.09 | Gingerglycolipid C |
| **$C_{33}H_{58}O_{14}$** | 678.3772 | 14.152 | 109454 | 474530 | 87.28 | Gingerglycolipid B |
| **$C_{42}H_{30}O_9$** | 678.1885 | 4.945 | 16024 | 56792 | 83.18 | alpha-Viniferin |
| **$C_{36}H_{58}O_{11}$** | 666.3939 | 16.002 | 84645 | 393522 | 88.61 | Quercilicoside A |
| **$C_{43}H_{64}O_5$** | 660.4724 | 8.573 | 36464 | 413546 | 91.13 | DG(22:6(4Z,7Z,10Z,13Z,16Z,19Z)/18:4(6Z,9Z,12Z,15Z)/0:0) |
| **$C_{36}H_{62}O_{10}$** | 654.4334 | 16.629 | 47407 | 115424 | 88.14 | Vinaginsenoside R10 |
| **$C_{36}H_{58}O_{10}$** | 650.4025 | 17.193 | 155843 | 638402 | 85.16 | Saponin H |

| | | | | | | |
|---|---|---|---|---|---|---|
| $C_{36}H_{54}O_{10}$ | 646.3695 | 16.155 | 38235 | 174209 | 85.45 | abrusoside A |
| $C_{35}H_{68}NO_7P$ | 645.4727 | 7.17 | 58199 | 644992 | 90.25 | PE(14:1(9Z)/P-16:0) |
| $C_{39}H_{51}NO_7$ | 645.3656 | 16.274 | 119794 | 1073156 | 94.07 | Janthitrem F |
| $C_{11}H_{22}O_{22}P_4$ | 629.9575 | 1.535 | 18495 | 75236 | 90.25 | Phosphatidylinositol-3,4,5-trisphosphate |
| $C_{28}H_{33}O_{16}$ | 625.1771 | 11.432 | 44834 | 316559 | 86.14 | Petunidin 3-rhamnoside 5-glucoside |
| $C_{28}H_{33}O_{15}$ | 609.1823 | 15.06 | 45915 | 275871 | 90.11 | Peonidin 3-rhamnoside 5-glucoside |
| $C_{30}H_{53}NO_{11}$ | 603.3627 | 18.861 | 83688 | 518503 | 91.75 | Fumonisin AK1 |
| $C_{35}H_{54}N_2O_6$ | 598.3975 | 5.296 | 104638 | 503114 | 92.97 | 3'-N-Acetyl-4'-O-(9-octadecenoyl)fusarochromanone |
| $C_{30}H_{28}O_{13}$ | 596.1579 | 7.764 | 74085 | 379945 | 90.31 | Eriodictyol 7-(6-trans-p-coumaroylglucoside) |
| $C_{26}H_{29}N_2O_{14}$ | 593.1725 | 13.966 | 44272 | 43403 | 90.9 | 2-Decarboxyphyllocactin |
| $C_{34}H_{56}O_8$ | 592.3991 | 15.455 | 79441 | 1651922 | 85.51 | Tuberoside |
| $C_{29}H_{36}O_{13}$ | 592.2151 | 18.032 | 2196637 | 10506288 | 87.88 | 8-Acetoxy-4'-methoxypinoresinol 4-glucoside |
| $C_{29}H_{36}O_{13}$ | 592.2178 | 17.86 | 1529307 | 13406469 | 85.13 | 8-Acetoxy-4'-methoxypinoresinol 4-glucoside |
| $C_{28}H_{30}O_{14}$ | 590.1642 | 14.802 | 56817 | 166182 | 85.27 | Maysin 3'-methyl ether |
| $C_{37}H_{48}O_6$ | 588.3456 | 19.714 | 84797 | 438794 | 91.21 | Hydratopyrrhoxanthinol |
| $C_{26}H_{30}O_{15}$ | 582.1607 | 7.346 | 25355 | 178789 | 89.84 | Asperuloside tetraacetate |
| $C_{38}H_{56}O_4$ | 576.4165 | 5.31 | 71958 | 314071 | 87.34 | 2-Hexaprenyl-3-methyl-5-hydroxy-6-methoxy-1,4-benzoquinone |
| $C_{37}H_{66}O_4$ | 574.5002 | 17.428 | 268925 | 2449230 | 92.63 | Montecristin |
| $C_{33}H_{28}O_9$ | 568.1732 | 4.245 | 45129 | 175152 | 91.31 | Asticolorin C |
| $C_{29}H_{58}NO_7P$ | 563.3949 | 18.924 | 149859 | 100375 | 90.3 | LysoPE(0:0/24:1(15Z)) |
| $C_{28}H_{34}O_{12}$ | 562.2036 | 6.188 | 44184 | 235913 | 86.76 | 5,7-Dihydroxy-3',4'-dimethoxy-8-(3-hydroxy-3-methylbutyl)-isoflavone 7-glucoside |
| $C_{35}H_{60}O_5$ | 560.4428 | 5.296 | 74401 | 330471 | 89.18 | Corepoxylone |
| $C_{24}H_{32}O_{15}$ | 560.1698 | 7.16 | 39055 | 132047 | 87.78 | 5''-(4-Hydroxy-(E)-cinnamoyl) alpha-L-arabinofuranosyl-(1->3)-beta-D-xylopyranosyl-(1->4)-D-xylopyranoside |
| $C_{33}H_{38}N_2O_5$ | 542.2764 | 18.34 | 1134745 | 4047772 | 90.44 | Chaetoglobosin N |
| $C_{25}H_{30}O_{13}$ | 538.1752 | 7.429 | 61102 | 542703 | 86.94 | Lippioside I |
| $C_{30}H_{47}NO_4S$ | 517.3251 | 13.495 | 244935 | 1241072 | 86.2 | Retapamulin |
| $C_{23}H_{39}N_3O_8$ | 485.2751 | 18.075 | 37970 | 139892 | 91.84 | Sarcodon scabrosus Depsipeptide |
| $C_{31}H_{48}O_4$ | 484.3531 | 15.525 | 64335 | 434271 | 87.32 | 3b,18b-3-Methoxy-11-oxo-12-oleanen-30-oic acid |
| $C_{30}H_{44}O_5$ | 484.3193 | 17.432 | 22380 | 73701 | 90.06 | Propapyriogenin A2 |
| $C_{21}H_{30}O_{12}$ | 474.1756 | 7.256 | 17044 | 119736 | 85.54 | 6-Feruloylglucose 2,3,4-trihydroxy-3-methylbutylglycoside |
| $C_{31}H_{42}O_3$ | 462.3136 | 16.646 | 72852 | 348161 | 91.16 | Hydroxysintaxanthin 5,6-epoxide |
| $C_{22}H_{20}O_{11}$ | 460.1005 | 1.618 | 43337 | 119003 | 86.59 | Glycitein 4'-O-glucuronide |
| $C_{20}H_{23}N_7O_6$ | 457.1726 | 9.542 | 13721 | 132294 | 90.29 | 5,10-Methylenetetrahydrofolate |
| $C_{18}H_{32}N_8O_6$ | 456.2372 | 17.684 | 1288131 | 14369689 | 90.12 | Rigin |
| $C_{22}H_{20}O_{10}$ | 444.1062 | 6.689 | 21041 | 409463 | 89.11 | Betavulgarin xyloside |
| $C_{23}H_{39}NO_5S$ | 441.2527 | 6.165 | 62219 | 283472 | 91.07 | Leukotriene E3 |
| $C_{29}H_{58}O_2$ | 438.4434 | 19.435 | 16368 | 45594 | 80.95 | Nonacosanoic acid |
| $C_{22}H_{42}O_8$ | 434.2882 | 7.177 | 25134 | 202413 | 90.23 | Polysorbate 60 |
| $C_{21}H_{36}O_9$ | 432.2306 | 15.415 | 97534 | 910459 | 92.35 | Glucosyl (2E,6E,10x)-10,11-dihydroxy-2,6-farnesadienoate |

| | | | | | | |
|---|---|---|---|---|---|---|
| **$C_{29}H_{46}O_2$** | 426.3471 | 12.546 | 27335 | 148159 | 88.86 | Stigmast-4-ene-3,6-dione |
| **$C_{19}H_{14}O_{11}$** | 418.053 | 1.525 | 55134 | 297886 | 86.6 | Shoyuflavone C |
| **$C_{23}H_{28}N_8$** | 416.2435 | 17.216 | 136891 | 1970985 | 97.39 | Forasartan |
| **$C_{29}H_{50}O$** | 414.3871 | 5.227 | 265994 | 1563669 | 85.96 | β-Sitosterol |
| **$C_{24}H_{48}NO_4$** | 414.3588 | 15.899 | 117382 | 19854 | 95.31 | Heptadecanoyl carnitine |
| **$C_{27}H_{40}O_3$** | 412.2962 | 12.218 | 108692 | 838456 | 88.79 | (9E)-Valenciaxanthin |
| **$C_{21}H_{30}O_8$** | 410.2015 | 8.079 | 59053 | 754502 | 88.12 | Scorzoside |
| **$C_{19}H_{34}O_9$** | 406.2174 | 4.593 | 205678 | 2446898 | 87.1 | Kiwiionoside |
| **$C_{19}H_{30}O_9$** | 402.189 | 2.169 | 9173 | 14927 | 86.41 | Methyl 7-epi-12-hydroxyjasmonate glucoside |
| **$C_{21}H_{23}NO_7$** | 401.1489 | 14.914 | 8422 | 43838 | 92.29 | Margrapine A |
| **$C_{24}H_{28}O_4$** | 380.1928 | 5.014 | 123041 | 831029 | 89.38 | Tokinolide A |
| **$C_{19}H_{29}N_3O_3S$** | 379.1897 | 5.021 | 83804 | 192503 | 87.61 | Thaumatin b, recombinant |
| **$C_{22}H_{26}O_5$** | 370.1764 | 5.532 | 115876 | 324788 | 92.89 | 3,17beta-Diacetoxyestra-1,3,5(10)-trien-6-one |
| **$C_{22}H_{44}O_2$** | 340.3343 | 14.881 | 18012 | 25750 | 88.47 | Docosanoic acid |
| **$C_{20}H_{41}NO_2$** | 327.3188 | 16.195 | 27259 | 166395 | 88.59 | N,N-Dimethylsphingosine |
| **$C_{18}H_{18}O_5$** | 314.117 | 18.042 | 8806 | 39015 | 88.28 | 6-Hydroxyenterolactone |
| **$C_{18}H_{14}O_5$** | 310.0844 | 5.492 | 17037 | 83652 | 88.91 | 6-Deoxyjacareubin |
| **$C_{15}H_{12}O_7$** | 304.0578 | 5.419 | 484823 | 2678026 | 87.22 | (±)-Taxifolin |
| **$C_{16}H_{28}N_2O_2$** | 280.2105 | 8.235 | 12571 | 59858 | 86.86 | Phygrine |
| **$C_{18}H_{19}NO$** | 265.146 | 7.028 | 43646 | 223054 | 89.45 | Mukoenine A |
| **$C_{18}H_{32}O$** | 264.2461 | 17.455 | 165784 | 1783055 | 96.13 | (±)-(Z)-2-(5-Tetradecenyl)cyclobutanone |
| **$C_9H_{12}O_7S$** | 264.0287 | 1.23 | 56189 | 146424 | 96.02 | 3-Methoxy-4-Hydroxyphenylglycol Sulfate |
| **$C_{15}H_{28}O_3$** | 256.2044 | 5.247 | 10892 | 12807 | 90.33 | Ipomeatetrahydrofuran |
| **$C_{12}H_{18}N_2O_4$** | 254.1247 | 7.631 | 41803 | 313464 | 87.09 | Midodrine |
| **$C_8H_{10}O_7S$** | 250.0123 | 0.746 | 5280 | 11554 | 95.87 | 3,4-Dihydroxyphenylglycol O-sulfate |
| **$C_{12}H_{24}N_2O_3$** | 244.1784 | 1.243 | 72724 | 96556 | 99.12 | N-(6-aminohexanoyl)-6-aminohexanoic acid |
| **$C_{12}H_{15}NO_4$** | 237.0999 | 7.688 | 40045 | 369318 | 87.09 | 3-Hydroxy-carbofuran |
| **$C_{16}H_{28}O$** | 236.2134 | 19.458 | 9137 | 20096 | 85.98 | Ambronide |
| **$C_{10}H_{20}N_2O_4$** | 232.143 | 7.664 | 37960 | 260081 | 93.8 | Isoleucyl-Threonine |
| **$C_9H_{17}N_3O_4$** | 231.1217 | 7.246 | 24558 | 207870 | 93.68 | Asparaginyl-Valine |
| **$C_{15}H_{26}O$** | 222.1982 | 11.183 | 26195 | 133933 | 88.18 | Faurinone |
| **$C_{13}H_{17}NS$** | 219.1078 | 6.049 | 28356 | 215913 | 86.03 | 2-Hexylbenzothiazole |
| **$C_{12}H_{22}O_3$** | 214.1577 | 12.583 | 18458 | 111416 | 87.67 | 4-Acetoxy-2-hexyltetrahydrofuran |
| **$C_5H_{12}NO_6P$** | 213.0405 | 5.97 | 33352 | 222527 | 82.66 | N-Lactoyl ethanolamine phosphate |
| **$C_{14}H_{28}O$** | 212.2113 | 13.429 | 46108 | 321939 | 86.32 | 12-Methyltridecanal |
| **$C_{13}H_{25}NO$** | 211.1924 | 16.48 | 9472 | 11014 | 85.66 | Ethyl menthane carboxamide |
| **$C_{15}H_{28}$** | 208.2178 | 15.465 | 12184 | 160728 | 86.14 | Cadinene |
| **$C_{14}H_{24}O$** | 208.1839 | 19.8 | 9400 | 41479 | 85.36 | 2-Decylfuran |
| **$C_{15}H_{22}$** | 202.1714 | 6.775 | 32932 | 60246 | 95.82 | beta-vatirenene |
| **$C_{10}H_{10}N_4O$** | 202.0859 | 18.214 | 36727 | 736458 | 85.28 | Metamitron |

| | | | | | | |
|---|---|---|---|---|---|---|
| $C_9H_{14}N_2O_3$ | 198.0997 | 1.532 | 429271 | 1944774 | 86.36 | Metharbital |
| $C_6H_8OS_2$ | 160.0022 | 18.423 | 8251 | 86561 | 87.27 | (2-Furanylmethyl) methyl disulfide |
| $C_6H_6N_2O_2$ | 138.0431 | 1.618 | 136387 | 469588 | 88.56 | para-Benzoquinone dioxime |
| $C_7H_7NO_2$ | 137.0473 | 7.094 | 15236 | 24829 | 91.35 | Salicylamide |
| $C_6H_{10}O_2$ | 114.0682 | 2.447 | 715759 | 7259424 | 87.12 | Allyl propionate |
| $C_6H_{14}O$ | 102.1045 | 18.423 | 318262 | 502355 | 94.75 | 3-Hexanol |
| $C_5H_{12}N$ | 86.0974 | 1.791 | 566325 | 768345 | 91.13 | Neurine |

**Table S3: Identified Compound from *Psidium guajava* extract using HR-ESI-MS.** The HR-ESI-MS data of *Psidium guajava* extract shows 40% of alkaloids (Peptide, Amino acids), 10% of Glycoside, 10% lipids, 10% of Phenolic compound (Aromatic Phenol, Quinone), and 10% of Terpenes, 15% of aliphatic Compounds (Fatty acids, alcohol and saturated, Unsaturated Alkenes) as well as 5% of other compounds were observed.

| Formula | Mass | RT | Area | Height | Score | Chemical Name |
|---|---|---|---|---|---|---|
| $C_{18}H_{18}N_2O_5$ | 342.1228 | 1.346 | 36328701 | 6341465 | 89.77 | Dictyoquinazol C |
| $C_{11}H_{23}N_2O_7PS$ | 358.0967 | 1.346 | 33637932 | 5795375 | 92.01 | D-Pantetheine 4'-phosphate |
| $C_{14}H_{20}O_{10}S$ | 380.0785 | 1.346 | 32567359 | 5651386 | 93.02 | 4-Methoxybenzyl O-(2-sulfoglucoside) |
| $C_{12}H_{14}O$ | 174.1042 | 1.313 | 5277170 | 2359957 | 86.12 | (R)-11,12,13-Trinor-1(5),6,9-guaiatrien-8-one |
| $C_{10}H_{16}O$ | 152.1211 | 1.376 | 4236221 | 942093 | 91.27 | alpha-Cyclocitral |
| $C_7H_{16}NO$ | 130.1233 | 1.134 | 785693 | 636421 | 93.51 | 4-Trimethylammoniobutanal |
| $C_{21}H_{40}O_9$ | 436.2684 | 14.928 | 4200628 | 778494 | 87.29 | (R)-1-O-[b-D-Apiofuranosyl-(1->2)-b-D-glucopyranoside]-1,3-octanediol |
| $C_{18}H_{35}N_3O_{13}$ | 501.2158 | 1.648 | 3215465 | 973292 | 90.38 | D-Glucosaminide |
| $C_6H_{14}O$ | 102.1048 | 18.284 | 254648 | 253617 | 93.23 | 3-Hexanol |
| $C_{42}H_{77}O_{10}P$ | 772.5257 | 17.495 | 3092491 | 338911 | 90.72 | PG(18:3(6Z,9Z,12Z)/18:0) |
| $C_{35}H_{56}O_7$ | 588.4054 | 12.636 | 1173809 | 345953 | 84.08 | 22-Angeloyltheasapogenol A |
| $C_{20}H_{30}O_{10}$ | 430.1826 | 19.001 | 2293200 | 396140 | 87.96 | Phenethyl rutinoside |
| $C_6H_8N_2O$ | 124.0629 | 5.18 | 42056 | 212958 | 87.08 | Methylimidazole acetaldehyde |
| $C_{13}H_{28}NO_2$ | 230.2103 | 6.218 | 392328 | 213161 | 86.07 | Capryloylcholine |
| $C_8H_{12}O$ | 124.0888 | 19.03 | 131368 | 37006 | 86.13 | trans, trans-3,5-Octadien-2-one |
| $C_{36}H_{72}NO_7P$ | 661.5047 | 18.642 | 2232877 | 459799 | 92.35 | PE(P-16:0/15:0) |
| $C_{29}H_{32}O_{13}$ | 588.1835 | 4.812 | 2728670 | 421837 | 88.22 | Etoposide |
| $C_{15}H_{17}NO_4$ | 275.1139 | 5.247 | 553250 | 123903 | 87.75 | 5-Amino-6-(4-hydroxy-2-butenoyl)-2,2-dimethyl-4- chromanone |
| $C_{12}H_{15}N_3O_6$ | 297.0958 | 5.247 | 552765 | 123005 | 95.83 | Musk xylene |
| $C_{42}H_{75}O_{10}P$ | 770.5104 | 15.362 | 606510 | 178893 | 91.34 | PG(18:3(9Z,12Z,15Z)/18:1(9Z)) |
| $C_{25}H_{50}NO_7P$ | 507.3329 | 6.265 | 888928 | 175901 | 85.77 | LysoPE(20:1(11Z)/0:0) |
| $C_{41}H_{62}O_2$ | 586.4755 | 15.147 | 2724165 | 201701 | 94.3 | 3',4'-Dihydrorhodovibrin |

| | | | | | | |
|---|---|---|---|---|---|---|
| $C_{26}H_{36}O_{13}$ | 556.2159 | 5.021 | 876596 | 187931 | 81.73 | 11-Hydroxyiridodial glucoside pentaacetate |
| $C_6H_8N_2O_2$ | 140.0588 | 4.806 | 1523345 | 208006 | 85.06 | 1,3-Dimethyluracil |
| $C_{42}H_{75}O_{10}P$ | 770.5094 | 14.994 | 122114 | 59904 | 88.93 | PG(18:3(9Z,12Z,15Z)/18:1(9Z)) |
| $C_{23}H_{38}O$ | 330.2907 | 12.59 | 925396 | 142637 | 89.02 | Teprenone |
| $C_{13}H_{18}N_2O_2$ | 234.136 | 9.25 | 2158813 | 164833 | 84.41 | Lenacil |
| $C_{28}H_{40}O_2$ | 408.3019 | 11.734 | 1305114 | 195772 | 86.57 | Ganodosterone |
| $C_{16}H_{25}NO_3$ | 279.183 | 6.553 | 1612887 | 162125 | 87.9 | Serratine |
| $C_{18}H_{35}NO_3$ | 313.2614 | 14.795 | 733022 | 147582 | 92.05 | (+)-Prosopinine |
| $C_{18}H_{24}O$ | 256.1811 | 15.001 | 1605689 | 129942 | 85.26 | Phenolic steroid |
| $C_{15}H_{22}N_2O_3$ | 278.1632 | 14.997 | 2101442 | 169261 | 92.61 | Isoleucyl-Phenylalanine |
| $C_{42}H_{75}O_{10}P$ | 770.5119 | 16.148 | 2129780 | 162690 | 83.76 | PG(18:3(9Z,12Z,15Z)/18:1(9Z)) |
| $C_{15}H_{19}NO_3$ | 261.1366 | 14.988 | 1766250 | 134516 | 91.44 | cis-1,3,4,6,7,11b-Hexahydro-9-methoxy-2H-benzo[a]quinolizine-3-carboxylic acid |
| $C_{22}H_{31}NO_3$ | 357.23 | 15.853 | 994179 | 76849 | 87.51 | Dihydroretrofractamide B |
| $C_6H_{14}NO_2$ | 132.1029 | 2.421 | 209487 | 40245 | 85.14 | beta-Alaninebetaine |
| $C_{22}H_{42}O_4$ | 370.3076 | 11.306 | 2135413 | 171477 | 87.28 | Di(2-ethylhexyl) adipate |
| $C_{15}H_{22}N_2O_3$ | 278.1636 | 15.313 | 1322964 | 159426 | 95.13 | Isoleucyl-Phenylalanine |
| $C_{21}H_{29}FO_4$ | 364.2035 | 11.339 | 668712 | 115939 | 86.74 | 6alpha-Fluoro-11beta,17-dihydroxypregn-4-ene-3,20-dione |
| $C_{24}H_{28}O_3$ | 364.2035 | 11.339 | 668712 | 115939 | 94.67 | 3-(1,1-Dimethyl-2-propenyl)-8-(3-methyl-2-butenyl)xanthyletin |
| $C_{21}H_{23}NO_5$ | 369.1588 | 11.339 | 858486 | 126597 | 86.73 | 7-Hydroxydehydroglaucine |
| $C_{14}H_{15}N$ | 197.1207 | 5.754 | 1219640 | 153120 | 94.12 | 2-(3-Phenylpropyl)pyridine |
| $C_{24}H_{36}O_8$ | 452.2402 | 1.774 | 130107 | 38500 | 85.47 | Rhodojaponin I |
| $C_{37}H_{64}O_8$ | 636.4612 | 12.573 | 1477672 | 199545 | 86.17 | 9-Oxoasimicinone |
| $C_{20}H_{12}$ | 252.0931 | 5.137 | 739252 | 135740 | 88.66 | Benzpyrene |
| $C_{43}H_{66}O_{16}$ | 838.4363 | 12.328 | 3571429 | 429579 | 92.21 | Talinumoside I |
| $C_{17}H_{12}O_4$ | 280.0726 | 4.593 | 762048 | 121373 | 93.1 | 3-Hydroxy-2-(4-methylbenzoyl)-4H-1-benzopyran-4-one |
| $C_{11}H_{23}N_3O_2$ | 229.1789 | 9.668 | 585172 | 96268 | 89.48 | n1,n8-diacetylspermidine |
| $C_{21}H_{23}NO_5$ | 369.1587 | 10.898 | 972916 | 99631 | 89.4 | 7-Hydroxydehydroglaucine |
| $C_{34}H_{38}N_4O_6$ | 598.2778 | 1.446 | 398273 | 72867 | 87.29 | Hematoporphyrin |
| $C_{38}H_{76}NO_7P$ | 689.536 | 18.626 | 371315 | 97293 | 88.77 | PE(15:0/P-18:0) |
| $C_9H_{12}FN_2O_9P$ | 342.0267 | 1.227 | 155814 | 107774 | 96.78 | 5-Fluorouridine monophosphate |
| $C_{35}H_{54}O_3$ | 522.4061 | 9.943 | 633677 | 67322 | 85.43 | Panaxydol linoleate |
| $C_{15}H_{22}N_2O_4$ | 294.1585 | 1.947 | 186551 | 52028 | 89.59 | Isoleucyl-Tyrosine |
| $C_{23}H_{28}O_8$ | 432.1779 | 1.28 | 98921 | 45449 | 91.77 | (2S,4S,6S)-2-[2-(4-Hydroxy-3-meyhoxyphenyl)ethyl]tetrahydro-6-(4,5-dihydroxy-3-methoxyphenyl)-2H-pyran-4-yl 4-acetate |
| $C_{12}H_{23}N_3O_6$ | 305.159 | 3.844 | 5408947 | 114969 | 86.95 | Syndesine |
| $C_{18}H_{19}NO$ | 265.1466 | 1.227 | 27834 | 63219 | 96.02 | Mukoenine A |
| $C_{22}H_{32}O_9$ | 440.205 | 1.496 | 842774 | 73812 | 87.25 | 3'-Hydroxy-HT2 toxin |
| $C_8H_{13}NS$ | 155.0759 | 1.234 | 895750 | 95467 | 87.95 | 4-Ethyl-5-propylthiazole |
| $C_{19}H_{27}NO_{11}$ | 445.159 | 1.313 | 134420 | 40229 | 85.36 | Lucuminamide |
| $C_{24}H_{35}NO_5$ | 417.2516 | 16.55 | 1437955 | 52978 | 85.9 | Batrachotoxinin A |

| | | | | | | |
|---|---|---|---|---|---|---|
| $C_{15}H_{26}O$ | 222.1985 | 1.237 | 132821 | 77578 | 87.79 | Faurinone (T) |
| $C_{10}H_{20}N_2O_3S$ | 248.1201 | 1.224 | 60498 | 88218 | 85.84 | Valyl-Methionine |
| $C_{17}H_{27}NO_2$ | 277.2032 | 14.533 | 1670288 | 56186 | 85.64 | Terbucarb |
| $C_{33}H_{42}O_{20}$ | 758.2306 | 4.587 | 1113213 | 127885 | 87.15 | Rubrofusarin 6-[glucosyl-(1->3)-glucosyl-(1->6)-glucoside] |
| $C_{39}H_{62}O_{15}$ | 770.4085 | 5.107 | 358696 | 85984 | 89.96 | Scopoloside II |
| $C_{27}H_{47}N_7O_6$ | 565.3591 | 19.007 | 388663 | 39100 | 86.26 | JSTX-3 |
| $C_{59}H_{90}O_8$ | 926.6661 | 5.92 | 152744 | 42573 | 88.68 | Thermozeaxanthin-13 |
| $C_{40}H_{56}O_2$ | 568.4286 | 11.25 | 516119 | 55290 | 84.29 | Deoxymyxol/ ((2'S)-Plectaniaxanthin) |
| $C_8H_{10}N_2O_4$ | 198.0638 | 4.444 | 308802 | 52505 | 86.18 | Mimosine |
| $C_{42}H_{48}N_4O_{16}$ | 864.3064 | 4.713 | 963411 | 155639 | 92.5 | Precorrin 2 |
| $C_6H_8OS_2$ | 160.0018 | 1.121 | 209032 | 53737 | 84.64 | (2-Furanylmethyl) methyl disulfide |
| $C_{14}H_{27}NO_{10}S_3$ | 465.0819 | 1.23 | 141751 | 83672 | 85.98 | Glucohesperalin |
| $C_5H_{15}N_4O_3P$ | 210.0882 | 1.257 | 89144 | 48372 | 84.72 | N4-Phosphoagmatine |
| $C_4H_{10}OS$ | 106.0449 | 1.117 | 135143 | 46014 | 90.53 | 3-(Methylthio)-1-propanol |
| $C_{15}H_{16}O_7$ | 308.0915 | 6.716 | 456251 | 50963 | 85.12 | Allamandin |
| $C_{15}H_{23}NO_9$ | 361.1358 | 1.509 | 211133 | 41362 | 88.48 | De-O-methylsimmondsin |
| $C_{39}H_{36}N_4O_7$ | 672.2583 | 8.225 | 808056 | 103426 | 96.82 | 4-[N-(p-Coumaroyl)serotonin-4''-yl]-N-feruloylserotonin |
| $C_{12}H_{18}O_2S$ | 226.1026 | 6.388 | 46404 | 27650 | 88.77 | 3-[(2-Methyl-3-furanyl)thio]-4-heptanone |
| $C_{17}H_{28}$ | 232.2192 | 12.294 | 157414 | 36670 | 90.36 | 5alpha-Gonane |
| $C_{21}H_{32}O_6$ | 380.221 | 11.303 | 410176 | 39811 | 84.27 | [6]-Gingerdiol 3,5-diacetate |
| $C_{13}H_{14}N_2O_7$ | 310.0799 | 5.286 | 537181 | 52436 | 97.64 | Acromelic acid A |
| $C_{38}H_{46}O_8$ | 630.3211 | 18.818 | 157871 | 40671 | 91.35 | (R)-6'-O-(4-Geranyloxy-2-hydroxycinnamoyl)-marmin |
| $C_{18}H_{29}D_5O_2$ | 287.2884 | 11.575 | 259092 | 41158 | 85.55 | Oleic acid (d5) |
| $C_{11}H_{14}N_2O_2$ | 206.1053 | 4.275 | 457291 | 51283 | 86.43 | Pheneturide |
| $C_{13}H_{16}O_2$ | 204.1141 | 6.394 | 50903 | 25120 | 85.61 | Isobutyl cinnamate |
| $C_{11}H_{11}NO_2$ | 189.0786 | 4.262 | 538896 | 60264 | 98.48 | 1,3-Dimethyl-6,8-isoquinolinediol) |
| $C_{20}H_{31}NO_2$ | 317.236 | 13.983 | 244326 | 30280 | 87.31 | 17beta-Hydroxy-4,17-dimethyl-4-azaandrost-5-en-3-one |
| $C_{12}H_{22}O_6$ | 262.1415 | 15.007 | 299934 | 26250 | 91.55 | Triethylene glycol diglycidyl ether (Aliphatic epo) |
| $C_{19}H_{24}N_7O_8P$ | 509.1423 | 1.678 | 60796 | 19199 | 88.38 | N6,N6,O-Tridemethylpuromycin-5'-phosphate |
| $C_7H_9O_3P$ | 172.0291 | 1.114 | 189927 | 45069 | 80.17 | Monomethyl phenylphosphonate |
| $C_{15}H_{15}N_3O_2$ | 269.1174 | 1.605 | 72593 | 36090 | 85.86 | Disperse Yellow 3 |
| $C_{15}H_{12}N_2O_2$ | 252.0909 | 1.605 | 64520 | 31759 | 85.86 | 2-Hydroxycarbamazepine |
| $C_{10}H_{12}$ | 132.0932 | 4.414 | 100924 | 24239 | 86.25 | Dicyclopentadiene |
| $C_{14}H_{18}N_2O_7$ | 326.1123 | 1.937 | 152580 | 47048 | 87.67 | Dinobuton |
| $C_{27}H_{34}O_8$ | 486.226 | 1.496 | 254266 | 56580 | 89.65 | Austalide I |
| $C_{19}H_{21}NO_5$ | 343.1408 | 1.456 | 125439 | 151603 | 86.94 | N-trans-Feruloyl-4-O-methyldopamine |
| $C_{24}H_{34}O$ | 338.2614 | 1.234 | 38636 | 33560 | 88.68 | 3-(2,4-Cyclopentadien-1-ylidene)-5alpha-androstan-17beta-ol |
| $C_{15}H_{20}O_9$ | 344.1104 | 1.681 | 141461 | 28881 | 80.86 | Domesticoside |
| $C_{10}H_{17}N_3O_2$ | 211.132 | 1.432 | 65295 | 36976 | 96.4 | Isouron |
| $C_{25}H_{48}O_2$ | 380.364 | 15.886 | 38661 | 25573 | 85.6 | 10,12-Pentacosanedione |

| | | | | | | |
|---|---|---|---|---|---|---|
| **$C_{13}H_{26}NO_4$** | 260.1858 | 11.552 | 112524 | 22891 | 86.33 | O-hexanoyl-R-carnitine |
| **$C_{26}H_{24}O_5$** | 416.1604 | 18.195 | 294458 | 32538 | 87.77 | Calophyllolide |
| **$C_7H_{16}N_4O_4$** | 220.118 | 14.6 | 140303 | 16035 | 96.52 | 1D-1-Guanidino-3-amino-1,3-dideoxy-scyllo-inositol |
| **$C_5H_5NOS$** | 127.0102 | 1.214 | 96682 | 41071 | 87.75 | 2-Acetylthiazole |
| **$C_{18}H_{19}NO_4$** | 313.1296 | 3.887 | 71266 | 26992 | 88 | Acetylcaranine |
| **$C_6H_{11}NO_2$** | 129.0797 | 1.605 | 121319 | 74780 | 84.89 | 2-Pyrrolidineacetic acid |
| **$C_{20}H_{39}NO$** | 309.3029 | 4.68 | 10308 | 7704 | 94.21 | N-Hexadecanoylpyrrolidine |
| **$C_5H_{11}O_5$** | 151.0606 | 1.631 | 82602 | 28630 | 90.23 | L-(+)-Arabinose |
| **$C_{22}H_{33}NO_8$** | 439.2206 | 16.211 | 452170 | 22429 | 90.52 | Parsonsine |
| **$C_{16}H_{21}NO$** | 243.1629 | 4.6 | 25266 | 16990 | 85.53 | Isobutylphendienamide |
| **$C_9H_{12}N_2$** | 148.0991 | 18.195 | 9027 | 17089 | 87.12 | Nornicotine |
| **$C_{20}H_{28}N_2O$** | 312.2197 | 12.805 | 34300 | 12498 | 96.37 | Fruticosonine |
| **$C_7H_{16}OS$** | 148.0917 | 18.38 | 2707 | 13897 | 89.13 | R-3-(Methylthio)-1-hexanol |
| **$C_6H_9N_5O_2$** | 183.0741 | 1.25 | 75765 | 27543 | 93.17 | 2,6-Diamino-4-hydroxy-5-N-methylformamidopyrimidine |
| **$C_{18}H_{19}N_2O_2$** | 295.1449 | 1.303 | 43355 | 22329 | 86.84 | Nigellidine |
| **$C_{20}H_{19}NO_3$** | 321.1359 | 3.963 | 71661 | 17940 | 87.49 | Acronycine |
| **$C_{25}H_{44}N_2$** | 372.3511 | 14.928 | 20335 | 22394 | 83.3 | Kurchessine |
| **$C_{45}H_{54}N_8O_{10}$** | 866.3915 | 4.272 | 67120 | 18982 | 87.96 | Pristinamycin IA |
| **$C_{23}H_{44}O_4$** | 384.3233 | 17.153 | 118058 | 13151 | 87.55 | MG(20:1(11Z)/0:0/0:0) |
| **$C_{20}H_{26}O_{12}$** | 458.1401 | 6.182 | 122230 | 17193 | 88.03 | 7-Hydroxy-4-methylphthalide O-[arabinosyl-(1->6)-glucoside] |
| **$C_{12}H_{11}NO$** | 185.0844 | 0.832 | 56431 | 19372 | 88.47 | 4-Hydroxydiphenylamine |
| **$C_{30}H_{24}O_{13}$** | 592.1199 | 4.812 | 1235671 | 304798 | 84.25 | Protoleucomelone |
| **$C_{11}H_{11}NO_3$** | 205.0724 | 10.835 | 152026 | 14061 | 87.04 | Swietenidin B |
| **$C_{11}H_{14}N_2O_3$** | 222.0993 | 10.852 | 228582 | 20860 | 86.86 | Glycyl-Phenylalanine |
| **$C_6H_8N_2O_2$** | 140.0589 | 4.739 | 659506 | 201486 | 98.28 | 1,3-Dimethyluracil |
| **$C_{28}H_{24}N_2O_7$** | 500.1579 | 17.223 | 323503 | 17216 | 88.22 | Orcein |
| **$C_4H_8N_4O_4$** | 176.0551 | 10.749 | 158477 | 14125 | 89.23 | Allantoic acid |
| **$C_{15}H_{16}$** | 196.1247 | 13.047 | 36196 | 14863 | 89.42 | 1,3-Diphenylpropane |
| **$C_4H_5O_7P$** | 195.9766 | 5.273 | 28131 | 6218 | 94.77 | 1-Carboxyvinyl carboxyphosphonate |
| **$C_9H_{15}NS$** | 169.0925 | 5.141 | 3404 | 6657 | 87.8 | 4-ethyl-5-methyl-2-propylthiazole |
| **$C_9H_{12}O_2S$** | 184.056 | 0.557 | 57219 | 13484 | 94.46 | (+/-)-3-[(2-methyl-3-furyl)thio]-2-butanone |
| **$C_4H_8NO_7P$** | 213.0037 | 5.273 | 45915 | 11984 | 94.46 | 4-Phospho-L-aspartate |
| **$C_{18}H_{10}$** | 226.0779 | 1.469 | 37715 | 18096 | 88.59 | Benzo[ghi]fluoranthene |
| **$C_{19}H_{27}FO_2$** | 306.1989 | 10.835 | 85657 | 12472 | 95.31 | 2alpha-Fluoro-17beta-hydroxyandrost-4-en-3-one |
| **$C_{32}H_{44}N_2O_8$** | 584.3099 | 15.266 | 131280 | 14337 | 90.74 | Lappaconitine |
| **$C_4H_5O_7P$** | 195.977 | 5.094 | 15068 | 5438 | 93.6 | 1-Carboxyvinyl carboxyphosphonate |
| **$C_{37}H_{38}N_2O_7$** | 622.2658 | 15.266 | 164052 | 16251 | 85.76 | O-Methylsomniferine |
| **$C_{23}H_{34}O_{14}$** | 534.1922 | 5.35 | 37259 | 16904 | 87.79 | • Isosyringinoside |
| **$C_4H_8NO_7P$** | 213.0052 | 5.482 | 11068 | 7672 | 98.27 | 4-Phospho-L-aspartate |
| **$C_2H_7N_3O$** | 89.0594 | 1.234 | 9867 | 10119 | 87.41 | N-(Aminomethyl)urea |

| | | | | | | |
|---|---|---|---|---|---|---|
| **$C_{11}H_{13}NO_3$** | 207.0898 | 3.519 | 5239 | 7204 | 85.42 | Cantleyine |
| **$C_7H_{11}NO$** | 125.0841 | 17.555 | 6019 | 8651 | 85.36 | 2-Ethyl-4,5-dimethyloxazole |
| **$C_{29}H_{54}O_2$** | 434.4121 | 13.764 | 27623 | 15115 | 85.17 | Ximenoylacetone |
| **$C_{14}H_{24}O$** | 208.1838 | 19.75 | 22472 | 9711 | 91.72 | 2-Decylfuran |
| **$C_{16}H_{30}O_7$** | 334.1988 | 10.56 | 25751 | 8365 | 89.5 | trans-p-Menthane-7,8-diol 8-glucoside |
| **$C_{11}H_{22}O$** | 170.1672 | 8.275 | 3727 | 7837 | 86.7 | 6-Undecanone |
| **$C_{17}H_{23}N_3O_3$** | 317.1728 | 10.56 | 28616 | 8531 | 85.23 | Isoleucyl-Tryptophan |
| **$C_{14}H_{10}F_4N_4O_7S$** | 454.0207 | 17.448 | 63392 | 8952 | 88.28 | Primisulfuron |
| **$C_{10}H_{12}N_2$** | 160.0994 | 11.558 | 62031 | 16110 | 84.73 | 2-(2-Aminoethyl)indole |
| **$C_{23}H_{38}O$** | 330.2899 | 12.324 | 641409 | 67984 | 85.62 | Teprenone |
| **$C_5H_7N$** | 81.0571 | 7.21 | 5781 | 6804 | 87.78 | 1-Methylpyrrole |
| **$C_{14}H_{20}N_4O_2$** | 276.1579 | 1.131 | 18059 | 11493 | 88.92 | p-Coumaroylagmatine |
| **$C_{14}H_{16}N_2O_8$** | 340.091 | 5.439 | 15016 | 7733 | 89.2 | Vulgaxanthin-II |
| **$C_{10}H_{12}N_5O_7P$** | 345.0466 | 5.439 | 14601 | 7621 | 85.71 | 2',3'-Cyclic GMP |
| **$C_7H_4O_7$** | 199.9971 | 19.883 | 13005 | 5262 | 90.91 | Poppy acid |
| **$C_{24}H_{40}N_2$** | 356.319 | 19.299 | 10861 | 10211 | 86.26 | Conessine |
| **$C_{14}H_{12}N_4O_2$** | 268.0962 | 9.8 | 3240 | 6159 | 85.09 | Disperse Blue 1 |
| **$C_{17}H_{20}O_5$** | 304.1316 | 2.762 | 4890 | 6777 | 87.86 | 3'-Deoxyoleacein |
| **$C_{10}H_{20}N_2O_4$** | 232.1412 | 10.225 | 10100 | 10361 | 85.55 | Isoleucyl-Threonine |
| **$C_{47}H_{86}O_{16}P_2$** | 968.5384 | 19.903 | 9958 | 4682 | 88.41 | PIP(18:2(9Z,12Z)/20:1(11Z) |
| **$C_6H_6O_5S$** | 189.9937 | 1.197 | 1917 | 3751 | 94.95 | 4-Sulfocatechol |
| **$C_{15}H_{19}NO_{10}$** | 373.1016 | 18.012 | 8810 | 2812 | 87.24 | DIMBOA-glucoside |
| **$C_{13}H_{27}N_3O_7$** | 337.1859 | 2.806 | 3713 | 8035 | 93.42 | Istamycin AO |
| **$C_{19}H_{20}O_{12}$** | 440.0915 | 1.804 | 2699 | 4277 | 90.33 | 3,5-Dihydroxyphenyl 1-O-(6-O-galloyl-beta-D-glucopyranoside) |
| **$C_{54}H_{109}NO_8P$** | 930.7885 | 17.972 | 5970 | 5481 | 89.66 | PC(22:0/24:0) |
| **$C_{11}H_{16}O_{10}$** | 308.0736 | 1.23 | 13396 | 8527 | 87.6 | D-Erythroascorbic acid 1'-a-D-glucoside |
| **$C_9H_{11}ClN_2O_2$** | 214.0508 | 8.361 | 238960 | 25930 | 85.53 | Monolinuron |
| **$C_{27}H_{33}NO_5$** | 451.2352 | 1.781 | 1763478 | 436445 | 77.56 | 14alpha-Hydroxypaxilline |

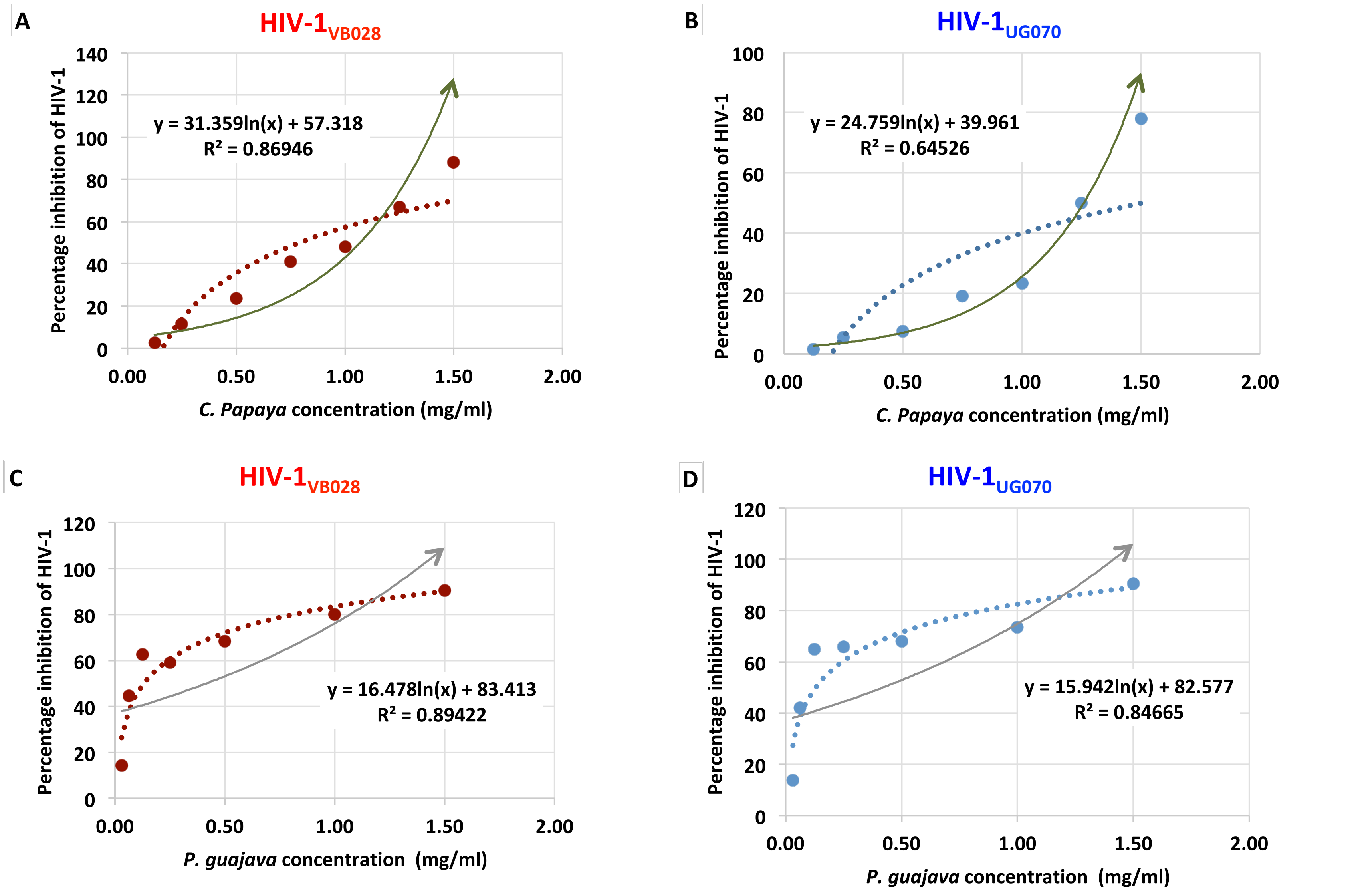


**Figure S1:** Anti-HIV-1 activity of *C. papaya* and *P. guajava* in cell associated study. Dose-dependent inhibition of (A) HIV-$1_{VB028}$ and (B) HIV-$1_{UG070}$ replication in presence of *C. papaya* extract (0.125-1.500mg/ml). The effect of different concentrations of *P. guajava* extract (0.03125-1.500mg/ml) on (C) HIV-$1_{VB028}$ and (D) HIV-$1_{UG070}$ replication.

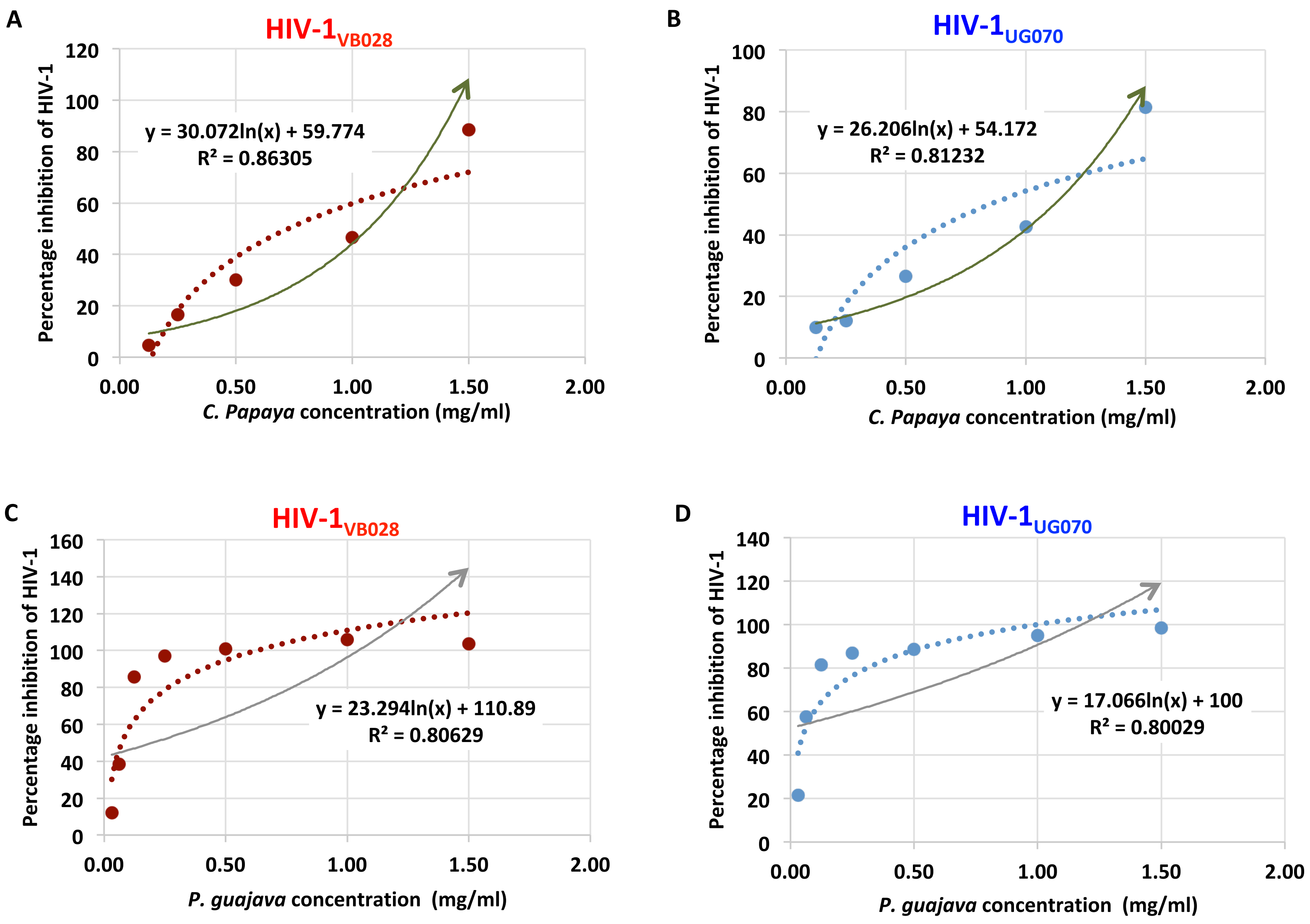


**Figure S2:** Effects of *C. papaya* and *P. guajava* on HIV-1 suppression through cell-free assays. Percentage of inhibition observed in dose dependent manner for (A) HIV-$1_{VB028}$ and (B) HIV-$1_{UG070}$ in presence of *C. papaya* extract (0.125-1500mg/ml). The effect of different concentrations of *P. guajava* extract (0.03125-1.500mg/ml) on (C) HIV-$1_{VB028}$ and (D) HIV-$1_{UG070}$ isolates.

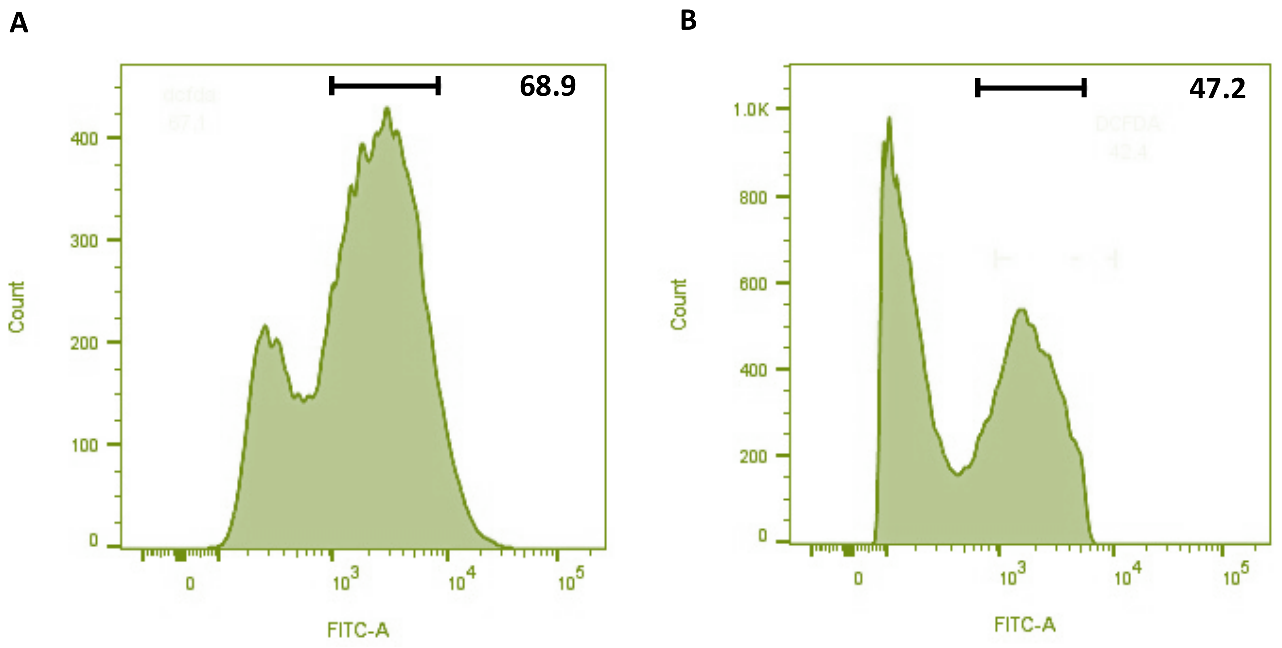


**Figure S3:** (A) ROS generator 15µM $H_2O_2$ ($H_2O_2$ generator) for 6 h served as positive control. (B) 15µM $H_2O_2$ ($H_2O_2$ generator) and 250U Catalase served as scavenger/inhibitor of ROS generation in the experiment.
